# Identification of recurrent fusion genes across multiple cancer types

**DOI:** 10.1038/s41598-019-38550-6

**Published:** 2019-01-31

**Authors:** Yan-Ping Yu, Peng Liu, Joel Nelson, Ronald L. Hamilton, Rohit Bhargava, George Michalopoulos, Qi Chen, Jun Zhang, Deqin Ma, Arjun Pennathur, James Luketich, Michael Nalesnik, George Tseng, Jian-Hua Luo

**Affiliations:** 1 Departments of Pathology, University of Pittsburgh School of Medicine, Pittsburgh, PA 15261 USA; 2 Departments of Biostatistics, University of Pittsburgh School of Medicine, Pittsburgh, PA 15261 USA; 3 Departments of Urology, University of Pittsburgh School of Medicine, Pittsburgh, PA 15261 USA; 4 Department of Pharmacology, Toxicology & Therapeutics, University of Kansas, Kansas City, KS 66160 USA; 5 Department of Medicine, University of Iowa, Iowa City, Iowa 52242 USA; 6 Department of Pathology, University of Iowa, Iowa City, Iowa 52242 USA; 7 Departments of Cardiothoracic Surgery, University of Pittsburgh School of Medicine, Pittsburgh, PA 15261 USA

## Abstract

Chromosome changes are one of the hallmarks of human malignancies. Chromosomal rearrangement is frequent in human cancers. One of the consequences of chromosomal rearrangement is gene fusions in the cancer genome. We have previously identified a panel of fusion genes in aggressive prostate cancers. In this study, we showed that 6 of these fusion genes are present in 7 different types of human malignancies with variable frequencies. Among them, the CCNH-C5orf30 and TRMT11-GRIK2 gene fusions were found in breast cancer, colon cancer, non-small cell lung cancer, esophageal adenocarcinoma, glioblastoma multiforme, ovarian cancer and liver cancer, with frequencies ranging from 12.9% to 85%. In contrast, four other gene fusions (mTOR-TP53BP1, TMEM135-CCDC67, KDM4-AC011523.2 and LRRC59-FLJ60017) are less frequent. Both TRMT11-GRIK2 and CCNH-C5orf30 are also frequently present in lymph node metastatic cancer samples from the breast, colon and ovary. Thus, detecting these fusion transcripts may have significant biological and clinical implications in cancer patient management.

## Introduction

In the last two decades, significant progress has been made in diagnosing and treating human cancers. However, cancers remain one of the most frequent causes of death in the United States. In 2017, 1,735,350 new cancer cases were diagnosed in the United States^1^. More than 600,000 cancer deaths are projected to occur in the United States in 2018: a death rate second only to cardiovascular diseases. Among human cancers, lung, prostate, breast, liver and colorectal cancers appear the most frequently, accounting for approximately 49% of all human cancers. These five types of cancers are projected to account for 305710 deaths in 2018 or over 50% of all cancer-related deaths in the US. Thus, understanding the mechanisms underlying the development of these cancers is crucial to reduce cancer mortality in the country.

Genome abnormalities are widely present in human cancers^2^. These abnormalities include single nucleotide mutations, copy number changes, chromosomal rearrangement, etc. Indeed, cancer genome abnormalities precede the development of cancer phenotypes^3–6^. Non-malignant tissues adjacent to cancers have been shown to contain similar genomic and transcriptomic changes as neighboring cancer tissues^3–11^. One of the salient abnormalities in the cancer genome is chromosomal rearrangements, which may result in the joining of 2 unrelated genes in the chromosome to produce a fusion gene. The most well-characterized example of fusion gene is the Philadelphia chromosome^12^ that joins the N-terminus of BCR with the tyrosine kinase domain of ABL^13^. The resulting chimeric protein has constitutively activated tyrosine kinase activity and transforms benign tissue into malignant one^14^. Several cancer-specific fusion genes have been discovered in prostate cancer samples^15–17^. Some of these fusion genes appear to be transforming^18,19^. Interestingly, one of the fusion genes, MAN2A1-FER, was found in 5 other types of human malignancies, and has been shown to transform normal livers into hepatocellular carcinomas in a short period of time^19^. SLC45A2-AMACR was found independently in bladder cancer^20^ and lung cancer cell lines^21^. These findings suggest that fusion genes may have wider implications than initially anticipated. To investigate whether fusion genes play a role in other human malignancies, we analyzed six fusion genes, including TRMT11-GRIK2, MTOR-TP53BP1, CCNH-C5orf30, KDM4-AC011523.2, TMEM135-CCDC67, and LRRC59-FLJ60017, in primary cancer samples from 7 different types of human malignancies and 20 cancer cell lines originating from 6 human cancers. These fusion genes are present in human cancers with variable frequencies, suggesting a much wider role for these cancer-specific fusion genes in the development of human malignancies.

## Materials and Methods

### Tissue samples

The 536 tissue specimens used in the study consist of 101 non-small cell lung cancers, 61 ovarian cancers, 60 colon cancers, 70 liver cancers, 150 glioblastoma, 60 breast cancers, and 34 esophageal adenocarcinomas. These samples were obtained from the University of Pittsburgh Tissue Bank in compliance with institutional regulatory guidelines (Supplemental Table 1 through 7). The informed consent exemptions and protocol were approved by the Institution Review Board of University of Pittsburgh. Cancer cells were obtained by macro-dissection. Esophageal cancer specimens were from a prospective IRB approved protocol from University of Pittsburgh and were frozen tissue samples. Sixteen non-small cell lung cancer samples were obtained from the University of Kansas. Twenty-eight non-small cell lung cancer samples were obtained from the University of Iowa. All informed consent exemptions and protocols were approved by the Institution Review Board of the University of Kansas or University of Iowa. All 20 cell lines used in the study were purchased from the American Type Cell Culture (ATCC, Inc., Manassas, VA, USA) and were cultured and maintained following the manufacturer’s recommendations.

### RNA extraction, cDNA synthesis and detection of fusion genes

Formalin-fixed paraffin-embedded (FFPE) tissue blocks of each sample were cut for multiple unstained slides. One of these slides was stained with hematoxylin and eosin. The cancer regions were circled by pathologists and macrodissected. Total RNA was extracted using trizol to lyse the cancer tissues (Invitrogen, CA). First strand cDNA was synthesized using ~2 µg of RNA from each sample, random hexamers and Superscript II^TM^ (Invitrogen, Inc, CA) at 42 °C for 2 hours. One microliter each cDNA sample was used for TaqMan PCR reactions with 50 heat cycles at 94 °C for 30 seconds, 61 °C for 30 seconds, and 72 °C for 30 seconds using primers and probes specific for CCNH-C5orf30 (AAAGTTATTTATCAGAGAGTCTGATGCTG/CTGTTCTACTCCAGGTATTTTCATTATATC; TaqMan probe, 5′-/56-FAM/ACAGGCAAG/ZEN/TTCTGTTCTCTTTCAGCA/3IABkFQ/-3′), mTOR-TP53BP1 (TGATAGACCAGTCCCGGGATG/CCACTGACATTCCCAGAACAAG; TaqMan probe, 5′-/56-FAM/TGTCAGCCT/ZEN/GTCAGAATCCAAGTCAAG/3IABkFQ/-3′), TRMT11-GRIK2 (GCGCTGTCGTGTACCCTTAAC/GAATGCAAGTTCCTCAGCTCC; TaqMan probe, 5′-/56-FAM/CGGAACTCC/ZEN/AGATGCTCCTGCG/3IABkFQ/-3′), LRRC59-FLJ60017 (GTGACTGCTTGGATGAGAAGC/CCCTCCTCTGGTTTGTTGTTG; TaqMan probe, 5′-/56-FAM/CAGTGTGCA/ZEN/AACAAGGTGACTGGAAG/3IABkFQ/-3′), TMEM135-CCDC67 (CAGCTGTCATGGAAGTTCAGAC/CCTCATTCTTTCCTGCTCAGAG; TaqMan probe, 5′-/56-FAM/AGTTCCTTT/ZEN/TAAGACTCACCAAGGGCAA/3IABkFQ/-3′), KDM4- AC011523.2 (AGACCACCTTCGCCTGGCAC/TCTCTCTCAGATCCAGGCTTG; TaqMan probe, 5′-/56-FAM/ACAGCATCA/ZEN/ACTACCTGCACTTTGGG/3IABkFQ/-3′), and β-actin (ACCCCACTTCTCTCTAAGGAG/GCAATGCTATCACCTCCCCTG; TaqMan probe, 5′-/56-FAM/CCAGTCCTC/ZEN/TCCCAAGTCCACAC/3IABkFQ/-3′) in a thermocycler (Eppendorf Realplex^TM^ thermocycler). A negative control and synthetic positive control were included in each batch of reactions. The PCR products were gel purified and Sanger-sequenced on 20% positive samples.

## Results

**Figure 1:**
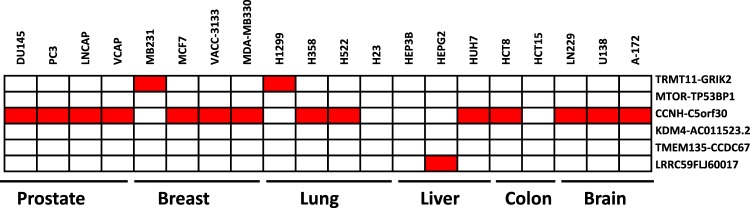
Fusion transcripts are present in human cancer cell lines. Representative cancer cell lines from the lung, liver, prostate, breast, colon and brain were examined for the presence of the TRMT11-GRIK2, CCNH-C5orf30, mTOR-TP53BP1, TMEM135-CCDC67, KDM4-AC011523.2 and LRRC59-FLJ60017 fusion transcripts using quantitative TaqMan RT-PCR. Red denotes positive detection of the fusion transcript, while blank indicates negative detection of the fusion transcript. All positive fusion samples were verified by Sanger sequencing (see Supplemental Figs 1–6).

In our previous studies^16,17^, we have characterized eight fusion genes identified in aggressive prostate cancer samples. Additional analyses showed that one of the fusion genes, MAN2A1-FER, is frequently present in 5 other types of human malignancies^19^. To expand our analyses of other fusion genes in the panel, we analyzed TRMT11-GRIK2, MTOR-TP53BP1, CCNH-C5orf30, KDM4-AC011523.2, TMEM135-CCDC67, and LRRC59-FLJ60017 in 20 cancer cell lines from six different human malignancies (Fig. 1). The TRMT11-GRIK2 fusion transcript was identified in the MD-MB-231breast cancer cell line and H1299 lung cancer cell line, while CCNH-C5orf30 was positive in 14 of 20 cancer cell lines, including all the prostate cancer cell lines tested (PC3, DU145, LNCaP and VCaP), 3 of 4 breast cancer cell lines (MCF7, VACC-3133 and MDA-MB330), 2 of 4 lung cancer cell lines (H358 and H522), 1 of 3 liver cancer cell lines (HepG2), 1 of 2 colon cancer cell lines (HCT8) and 3 of 3 GBM cell lines (LN229, U138 and A-172). LRRC59-FLJ60017 was also present in the HepG2 liver cancer cell line. These results suggest that these fusion genes are not specific for prostate cancer and may be present in primary cancer samples from a variety of human malignancies.

**Figure 2:**
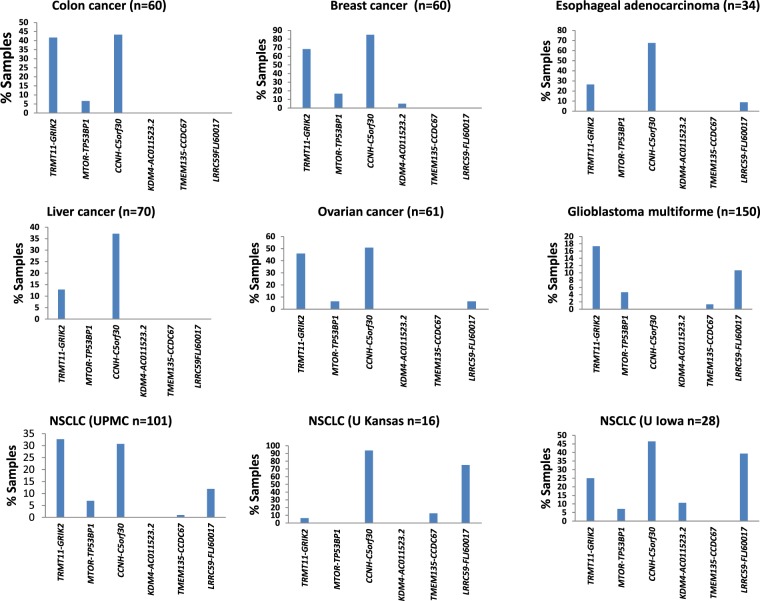
Frequency of gene fusion in primary human malignant samples. Real-time TaqMan qRT-PCR reactions were performed on formalin-fixed paraffin-embedded samples from breast cancer, colon cancer, esophageal adenocarcinoma, ovarian cancer, hepatocellular carcinoma, non-small cell lung cancer and glioblastoma multiforme. The number of samples from each cohort are indicated. Sanger sequencing was performed on 20–100% of samples that were found to be positive for the fusion genes (see Supplemental Figs 1–6).

To investigate whether any of the above-mentioned fusion genes has a role in human cancers, quantitative TaqMan qRT-PCR reactions using primers and probes specific for each fusion gene were performed on primary human cancer samples representing seven different types of human malignancies. Our results showed that TRMT11-GRIK2 is present in all seven types of human malignancies (Table 1, Fig. 2 and Supplemental Figs 1–6)), including breast cancer (41/60, 68.33%), colon cancer (25/60, 41.7%), esophageal adenocarcinoma (9/34, 26.5%), hepatocellular carcinoma (9/70, 12.9%), ovarian adenocarcinoma (28/61, 45.9%), glioblastoma multiforme (26/150, 17.3%) and non-small cell lung cancer (39/141, 27.7%). The CCNH-C5orf30 transcript was also detected in 7 different types of human cancers with the following relatively high frequencies: 85% (51/60) in breast cancer, 43% (26/60) in colon cancer, 50.8% (31/61) in ovarian cancer, 67.6% (23/34) in esophageal adenocarcinoma, 41.8% (59/141) in non-small cell lung cancer, 37% (26/70) in liver cancer and 53% (80/150) in glioblastoma multiforme. In contrast, mTOR-TP53BP1was only detected in five different types of human malignancies and with significantly lower frequencies, including breast cancer (10/60, 16.7%), colon cancer (4/60, 6.7%), ovarian adenocarcinoma (4/61, 6.6%), glioblastoma multiforme (7/150, 4.7%) and lung cancer (8/141, 5.7%). LRRC59-FLJ60017 was present in four different types of human cancers, esophageal adenocarcinoma (3/34, 8.8%), ovarian adenocarcinoma (4/61, 6.6%), glioblastoma multiforme (16/150, 10.7%), and non-small cell lung cancer (33/141, 23.4%). Only two glioblastoma multiforme and one lung cancer samples were positive for the TMEM135-CCDC67 fusion gene. The KDM4-AC011523.2 fusion was only found in 3 breast cancer and 3 lung cancer samples.

**Table 1:** Distribution of fusion genes in colon cancer, breast cancer, esophageal adenocarcinoma, liver cancer, ovarian adenocarcinoma, glioblastoma multiforme and non-small cell lung cancer.

Cancer	TRMT11-GRIK2 MTOR-TP53BP1 CCNH-C5orf30 KDM4-AC011523.2 TMEM135-CCDC67 LRRC59-FLJ60017					
**Breast cancer**						
Ductal Type	21/30	4/30	26/30	0/30	0/30	0/30
*With LN met*	7/15	0/15	11/15	0/15	0/15	0/15
*Without LN met*	14/15	4/15	15/15	0/15	0/15	0/15
Lobular	20/30	6/30	25/30	3/30	0/30	0/30
*With LN met*	12/15	0/15	10/15	0/15	0/15	0/15
*Without LN met*	8/15	6/15	15/15	3/15	0/15	0/15
Non-recurrence	36/53	10/53	47/53	3/53	0/53	0/53
Recurrence	5/7	0/7	4/7	0/7	0/7	0/7
Stage 1	21/33	8/33	30/33	3/33	0/33	0/33
Stage 2	11/16	2/16	12/16	0/16	0/16	0/16
Stage 3	6/9	0/9	7/9	0/9	0/9	0/9
Stage 4	1/2	0/2	2/2	0/2	0/2	0/2
Grade 1	5/8	2/8	8/8	0/8	0/8	0/8
Grade 2	26/38	8/38	33/38	3/38	0/38	0/38
Grade 3	10/14	0/14	10/14	0/14	0/14	0/14
ER positive	34/51	9/51	45/51	3/51	0/51	0/51
ER negative	7/9	1/9	6/9	0/9	0/9	0/9
PR positive	29/44	7/44	39/44	3/44	0/44	0/44
PR negative	12/16	3/16	12/16	0/16	0/16	0/16
HER2 amp+	2/3	0/3	3/3	0/3	0/3	0/3
HER2 amp−	39/57	10/57	48/57	3/57	0/57	0/57
Triple negative	7/9	1/9	6/9	0/9	0/9	0/9
**Colon cancer**						
With LN met	14/30	3/30	15/30	0/30	0/30	0/30
Without LN met	11/30	1/30	11/30	0/30	0/30	0/30
Recurrence 5-yr	3/5	1/5	4/5	0/5	0/5	0/5
Non-recurrence	22/55	3/55	22/55	0/55	0/55	0/55
Stage 1	1/3	0/3	1/3	0/3	0/3	0/3
Stage 2	3/13	0/13	2/13	0/13	0/13	0/13
Stage 3	20/42	3/42	21/42	0/42	0/42	0/42
Stage 4	1/2	1/2	2/2	0/2	0/2	0/2
Grade I	1/2	0/2	1/2	0/2	0/2	0/2
Grade II	19/51	3/51	21/51	0/51	0/51	0/51
Grade III	5/7	1/7	4/7	0/7	0/7	0/7
**Ovarian cancer**						
With LN met	14/30	3/30	18/30	0/30	0/30	2/30
Without LN met	14/31	1/31	13/31	0/31	0/31	2/31
Non-recurrence	16/33	1/33	15/33	0/33	0/33	2/33
Recurrence 5-yrs	12/28	3/28	16/28	0/28	0/28	2/28
Survive >5 yrs	16/34	1/34	18/34	0/34	0/34	2/34
Death from Ca	12/27	3/27	13/27	0/27	0/27	2/27
Stage 1	11/22	1/22	9/22	0/22	0/22	2/22
Stage 2	2/4	0/4	1/4	0/4	0/4	0/4
Stage 3	15/35	3/35	21/35	0/35	0/35	2/35
Grade I	5/7	1/7	2/7	0/7	0/7	0/7
Grade II	2/8	0/8	4/8	0/8	0/8	0/8
Grade III	16/34	2/34	16/34	0/34	0/34	3/34
Grade IV	5/12	1/12	9/12	0/12	0/12	1/12
**Esophageal Adenocarcinoma**						
	9/34	0/34	23/34	0/34	0/34	3/34
**Non-small cell lung cancer**						
UPMC cohort	33/101	7/101	31/101	0/101	1/101	12/101
Stage 1	10/24	3/24	8/24	0/24	0/24	3/24
Stage 2	19/62	4/62	21/62	0/62	1/62	7/62
Stage 3	2/5	0/5	0/5	0/5	0/5	0/5
Stage 4	2/10	0/10	2/10	0/10	0/10	2/10
With LN met	10/39	2/39	12/39	0/39	0/39	5/39
Without LN met	23/62	5/62	19/62	0/62	1/62	7/62
Survival 5-yr	9/34	2/34	10/34	0/34	0/34	6/34
Death 5 yrs	22/67	5/67	21/67	0/67	1/67	6/67
Ad Ca	0/0	0/0	0/0	0/0	0/0	0/0
Sq Ca	33/101	7/101	31/101	0/101	1/101	12/101
Kansas cohort	1/16	0/16	15/16	0/16	2/16	12/16
Stage I	0/5	0/5	5/5	0/5	0/5	4/5
Stage II	1/11	0/11	10/11	0/11	2/11	8/11
Ad Ca	1/8	0/8	8/8	0/8	0/8	6/8
Sq Ca	0/8	0/8	7/8	0/8	2/8	6/8
Iowa cohort	5/24	1/24	13/24	3/24	0/24	9/24
Stage I	2/8	0/8	4/8	0/8	0/8	3/8
Stage II	2/7	1/7	5/7	2/7	0/7	5/7
Stage III	0/6	0/6	3/6	1/6	0/6	1/6
Stage IV	1/3	0/3	1/3	0/3	0/3	0/3
With met	3/16	1/16	7/16	2/16	0/16	6/16
Without met	2/8	0/8	6/8	1/8	0/8	3/8
Ad Ca	5/23	1/23	13/23	3/23	0/23	9/23
Sq Ca	0/0	0/0	0/0	0/0	0/0	0/0
Sarcomatoid	0/1	0/1	0/1	0/1	0/1	0/1
**Liver cancer**						
Total	9/70	0/70	26/70	0/70	0/70	0/70
Survive 5 yrs	5/12	0/12	7/12	0/12	0/12	0/12
Death	4/58	0/58	19/58	0/58	0/58	0/58
Non-recurrence	5/34	0/34	13/34	0/34	0/34	0/34
Recurrence	4/36	0/36	13/36	0/36	0/36	0/36
**Glioblastoma multiforme**						
	26/150	7/150	80/150	0/150	2/150	16/150

Interestingly, ductal type breast cancers positive for TRMT11-GRIK2 were associated with a lower likelihood of developing local lymph node metastasis (46.7% versus 93.3%, p = 0.014). Liver cancers positive for TRMT11-GRIK2 were also associated with a higher rate of overall survival (41.7% versus 6.9%, p = 0.006). KDM4-AC011523.2 was only detected in lobular type breast cancer and adenocarcinoma of the lung. Patients with lobular breast cancers positive for mTOR-TP53BP1 were also less likely to have lymph node metastasis (0% versus 40%, p = 0.017). CCNH-C5orf30 was more frequent in lung cancer adenocarcinomas versus squamous type (67.7% versus 34.8%, p = 0.001) and colon cancer at advanced stages at the time of diagnosis (52.3% versus 18.8%, p = 0.037).

**Figure 3:**
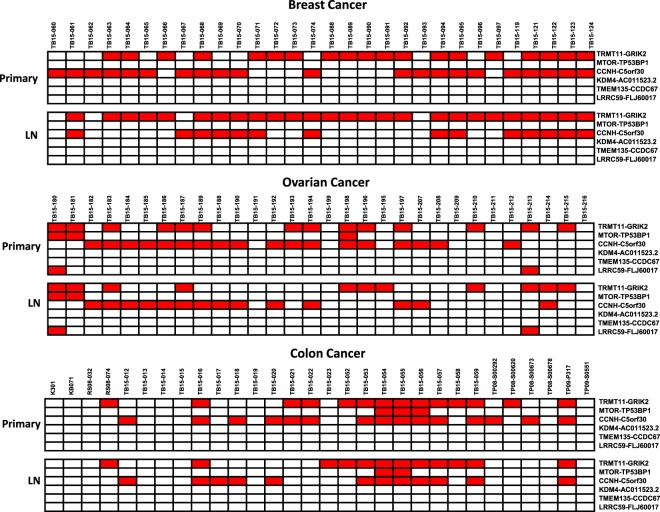
Fusion transcripts in metastatic lymph node samples. Real-time TaqMan qRT-PCR reactions were performed on formalin-fixed paraffin-embedded samples from breast cancer (top), ovarian cancer (middle) and colon cancer (bottom) and their corresponding lymph node metastases. Both primary (top) and matched lymph node (bottom) cancer samples are shown. Red indicates positive detection of the fusion transcript, while blank indicates negative detection of the fusion transcript. Sanger sequencing was performed on 20–100% of samples that were found to be positive for the fusion genes (see Supplemental Figs 1–6).

To investigate whether fusion genes are also present in human cancer metastatic lesions, breast cancer, colon cancer and ovarian cancer samples with matched lymph node metastases were analyzed (Fig. 3). Twenty-six of 30 metastatic breast cancers in lymph nodes were positive for TRMT11-GRIK2, including seven metastatic cancers whose matched primary breast cancers were negative for the fusion. For colon cancers, the matched status of TRMT11-GRIK2 between primary cancer samples and lymph node metastases was 78.5% (11/14Eleven lymph node metastases were exactly matched with the status of the primary colon cancer samples, while two samples of lymph node metastases were found negative for TRMT11-GRIK2 fusion. One lymph node metastasis was found positive for the fusion gene while the matched primary sample was negative (Fig. 3). For ovarian adenocarcinomas, nine metastatic lesions were found to contain the TRMT11-GRIK2 fusion gene, matching all the primary samples. However, four lymph node metastases contained no TRMT11-GRIK2 fusion gene while the matched primary cancer samples were positive. One lymph node metastasis gained the TRMT11-GRIK2 fusion over the primary cancer sample. For CCNH-C5orf30, the matching rate of primary breast cancer with lymph node metastases was 62%, while the matching rates for ovarian cancer and colon cancer with their corresponding lymph node metastases were 72% and 73%, respectively. For mTOR-TP53BP1, two of 3 lymph node metastases retained the fusion in colon and ovarian cancers. Additionally, two of 2 ovarian cancer lymph node metastases retained the LRRC59-FLJ60017 fusion. These results suggest significant heterogeneity among the cancer samples. However, most fusion genes were retained in metastatic lesions.

## Discussion

Gene fusions are the result of recombination of two unrelated genes. Fusion events can also involve genes that have similar biological roles (for example, between two genes that both have roles in transcription, such as the ESR1-YAP1 driver fusion in breast cancer)^22^. Almost all cancer-specific gene fusions are the result of chromosomal rearrangements or translocations^17^. There is increasing evidence suggesting that gene fusions are some of the key drivers of human cancer development. New gene fusion events have been discovered in prostate cancer^15–17^, breast cancer^23–25^, NSCLC^19,26,27^, colon cancer^28–30^, glioblastoma multiforme^19,31,32^, liver cancer^19,33–35^ and ovarian cancer^19,36,37^. Some of these fusion genes appear to play driver roles in the aggressive behaviors of these cancers^19,23,26,31^. Gene fusions produce two possible outcomes for the genes involved. One outcome is a gain of function due to the loss of the regulatory domain in the protein, so the enzymatic domain of the same protein becomes hyper-activated. BCR-ABL and MAN2A1-FER are examples of gain of function fusion genes, leading to hyper-activation of the fusion partner. In-frame fusions can also contribute to disease pathogenesis by creating a fusion protein that contains not only complementary functions encoded by each partner gene, but also has neomorphic properties. ESR1-YAP1^22^, a driver fusion found in advanced breast cancer, generates a hyperactive transcription factor through the combination of the ESR1 part that provides domains necessary for DNA binding, dimerization, and nuclear localization and the YAP1 part that provides components for transcriptional activation. ESR1-YAP1 is able to drive expression of genes that promote metastatic biology, a function that full length wild-type ESR1 lacks. The other outcome is a loss of function due to the truncation of the head gene and/or complete elimination of the open reading frame of the tail gene, such as with TRMT11-GRIK2 and mTOR-TP53BP1. For both these instances, tumor suppressor activities of GRIK2^38^ and TP53BP1^39^ are lost due to the loss of protein translation.

Among the prostate cancer fusion genes identified in our previous study^16^, two of these fusion genes (SLC45A2-AMACR and MAN2A1-FER) were also found in other types of human cancers^19–21^, suggesting that these gene fusions are not specific to prostate cancer but may be widely present in human cancers. MAN2A1-FER was found to be the driver for liver cancer in mouse^19^. The expression of this fusion gene induced spontaneous liver cancer in mice by ectopically phosphorylating the EGFR extracellular domain and activating its signaling pathways^19^. Although the biological roles of the other fusion genes remain unknown, five of these gene fusion events (TMEM135-CCDC67, mTOR-TP53BP1, LRRC59-FLJ10067, KDM4-AC011523.2 and TRMT11-GRIK2) eliminate the open-reading frames in the tail genes and thus produce gene knockouts. CCDC67^40^, TP53BP1^39,41,42^ and GRIK2^38^ are tail genes that contain tumor suppressor activity. The TMEM135-CCDC67, mTOR-TP53BP1 and TRMT11-GRIK2 gene fusions are equivalent to the functional deletion of CCDC67, TP53BP1 and GRIK2, respectively. Deletions of these genes have been shown to promote the aggressive behaviors of cancers^38–42^. These gene fusion events may have significant biological implications in the development of cancer.

Based on the current analyses, TRMT11-GRIK2 and CCNH-C5orf30 are probably some of the most widely distributed gene fusions in human malignancies, being present in at least eight types of human malignancies. TRMT11-GRIK2 has frequencies ranging from 12.9% in liver cancer to 68.3% in breast cancer. This fusion gene is also found in a breast cancer cell line and a lung cancer cell line. CCNH-C5orf30 is also very frequent among different types of cancers and their corresponding cancer cell lines. In contrast, the positive rates of the other four fusion genes are much less frequent. The mechanism underlying the disparity of frequencies of these fusion genes is not clear. Although there is some correlation between the distance between the partner genes and the frequencies (both mTOR-TP53BP1 and LRRC59-FLJ60017 gene fusions have their gene partners located in different chromosomes, while both TRMT11-GRIK2 and CCNH-C5orf30 are located in the same chromosome with distances less than 24 MB), TMEM135 and CCDC67 are only separated by 6 MB, and the TMEM135-CCDC67 fusion is exceeding rare in cancers. Nevertheless, the presence of these gene fusions suggests that chromosomal recombination and translocation are probably some of the most frequent events in human cancers.

TRMT11 is a tRNA methyltransferase. The protein is essential for m^2^G formation at position 10 in tRNA^43^. This methylation event is required for tRNA stability and translation activity^44^. In contrast, GRIK2 encodes a glutamate receptor^45^ and was shown to possess tumor suppressor activity^38^. The process of chromosomal recombination between TRMT11 and GRIK2 to create the TRMT11-GRIK2 gene fusion destroys the open-reading frames of both genes and produces functional knockouts of these two proteins. The absence of TRMT11 may produce less efficient and unstable translation of mRNA into protein in cancer cells due to tRNA defects. Alternatively, protein translation may be repressed. The lack of GRIK2 may accelerate cell cycle progression and promote cell migration. Thus, cells with the TRMT11-GRIK2 gene fusion may be unstable and tumorigenic.

CCNH is an important member of the cyclin family. It complexes with cdk7-MAT1 and is a component in the TFIIH and RNA polymerase complexes^46,47^. Thus, CCNH is a critical regulator for the processes of transcription and cell cycle progression. The CCNH-C5orf30 gene fusion produces a truncated cyclin H protein with the deletion of its H5′ and HC domains. A study showed that CCNH mutants lacking the HC domain do not activate cdk7^47^. As a result, the truncated CCNH from the gene fusion may have a negative impact on the functions of RNA polymerase and TFIIH. C5orf30 was also shown to inhibit the generation of cytokines involved in inflammation, such as TNF and IL1, and promote the expression of anti-inflammatory cytokines, such as IL10, in rheumatoid arthritis synovial fibroblasts^48,49^. The CCNH-C5orf30 fusion transcript contains an intact C5orf30 opening reading frame. The CCNH-C5orf30 gene fusion places C5orf30 expression under the CCNH promoter and may promote its expression. Over-expression of C5orf30 in cancer cells may help to fend off immune responses targeting the cancers.

The wide presence of the 6 fusion genes in a variety of human malignancies may provide significant utility for clinical cancer diagnosis and therapeutic targeting. The presence of these fusion genes in metastatic cancer samples can be used in clinical follow-up studies to analyze the recurrence of human cancers. If these fusion genes are present in grey zone biopsy samples, it may also help to confirm or to make a correct diagnosis. Furthermore, the chromosomal breakpoints of significant numbers of these fusion genes have been identified. These chromosome breakpoints not only serve as cancer markers but also provide unique opportunities to treat human cancers using genome editing technologies^50^. When multiple fusion gene breakpoints are present in the same cancer cells, multi-targeting at these chromosomal breakpoints may significantly enhance the efficiency of genome editing treatments targeting the cancer cells.

## Supplementary information

Supplemental tables 1-7, supplemental figure legend and supplemental figures 1-6
