# Supplementary material for: Identification of recurrent fusion genes across multiple cancer types
Source: Sci Rep. 2019 Jan 31;9:1074. doi: 10.1038/s41598-019-38550-6 (PMC6355770; doi:10.1038/s41598-019-38550-6)

Identification of recurrent fusion genes across multiple cancer types

By

Yan-Ping Yu, Peng Liu, Joel Nelson, Ronald L. Hamilton, Rohit Bhargava, George Michalopoulos, Qi Chen, Jun Zhang, Deqin Ma, Arjun Pennathur, Michael Nalesnik, George Tseng and Jian-Hua Luo

Supplemental table 1 Breast Cancer

Case	TRMT11-GRIK2	MTOR-TP53BP1	CCNH-C50RF30	KDM4-AC011523.2	TMEM135-CCDC67	LRRC59-FLJ60017	Age	Histo	Grade	pT	pN	pM	ER Assay	PR Assay	HER2	Recurrence	Months to Recurrence	Survival In Months
TB15-060			P				38	Ductal carcinoma	Grade I:	3	1	0	Positive/Elevated	Positive/Elevated	Negative/Normal	No	N/A	067
TB15-061			P				59	Ductal carcinoma	Grade II:	1C	1	0	Positive/Elevated	Positive/Elevated	Negative/Normal	No	N/A	074
TB15-062			P				50	Ductal carcinoma	Grade II:	2	1M	X	Positive/Elevated	Positive/Elevated	Negative/Normal	No	N/A	062
TB15-063	P		P				54	Ductal carcinoma	Grade II:	1C	1A	0	Positive/Elevated	Negative, Normal	Negative/Normal	No	N/A	061
TB15-064	P		P				49	Ductal carcinoma	Grade II:	2	1M	0	Positive/Elevated	Positive/Elevated	Negative/Normal	No	N/A	061
TB15-065			P				28	Ductal carcinoma	Grade III	1C	2A	0	Positive/Elevated	Positive/Elevated	Negative/Normal	No	N/A	060
TB15-066	P						55	Ductal carcinoma	Grade II:	1C	1A	X	Positive/Elevated	Negative, Normal	Negative/Normal	No	N/A	059
TB15-067			P				64	Ductal carcinoma	Grade II:	2	1A	0	Negative, Normal	Negative, Normal	Negative/Normal	No	N/A	068
TB15-068	P		P				61	Ductal carcinoma	Grade III	3	3B	X	Positive/Elevated	Negative, Normal	Negative/Normal	Yes	018	028
TB15-069			P				71	Ductal carcinoma	Grade II:	1B	1A	X	Positive/Elevated	Positive/Elevated	Negative/Normal	No	N/A	063
TB15-070			P				45	Ductal carcinoma	Grade III	3	2A	X	Negative, Normal	Negative, Normal	Negative/Normal	Yes	027	037
TB15-071	P						41	Ductal carcinoma	Grade III	3	2A	0	Negative, Normal	Negative, Normal	Negative/Normal	Yes	020	023
TB15-072	P						63	Ductal carcinoma	Grade III	2	1C	X	Negative, Normal	Negative, Normal	Negative/Normal	Yes	013	018
TB15-073	P						79	Ductal carcinoma	Grade III	1C	1A	0	Negative, Normal	Negative, Normal	Negative/Normal	No	N/A	060
TB15-074			P				27	Ductal carcinoma	Grade III	1A	1A		Positive/Elevated	Positive/Elevated	Negative/Normal	No	N/A	063
TB15-076	P		P				67	Ductal carcinoma	Grade II:	1B	0	0	Positive/Elevated	Positive/Elevated	Negative/Normal	No	N/A	061
TB15-077	P		P				62	Ductal carcinoma	Grade II:	1A	0	X	Positive/Elevated	Positive/Elevated	Negative/Normal	No	N/A	064
TB15-078	P		P				64	Ductal carcinoma	Grade III	1C	0	0	Negative/Normal	Negative/Normal	Negative/Normal	No	N/A	065
TB15-079		P	P				76	Ductal carcinoma	Grade I:	1B	0	0	Positive/Elevated	Positive/Elevated	Negative/Normal	No	N/A	060
TB15-080	P	P	P				67	Ductal carcinoma	Grade II:	1B	0	0	Positive/Elevated	Positive/Elevated	Negative/Normal	No	N/A	057
TB15-081	P	P	P				57	Ductal carcinoma	Grade II:	1C	0	0	Positive/Elevated	Positive/Elevated	Negative/Normal	No	N/A	058
TB15-082	P		P				45	Ductal carcinoma	Grade II:	2	0	X	Positive/Elevated	Positive/Elevated	Negative/Normal	No	N/A	063
TB15-083	P		P				38	Ductal carcinoma	Grade I:	2	0	0	Positive/Elevated	Positive/Elevated	Negative/Normal	No	N/A	062
TB15-084	P	P	P				68	Ductal carcinoma	Grade I:	1B	0	0	Positive/Elevated	Positive/Elevated	Negative/Normal	No	N/A	060
TB15-085	P		P				58	Ductal carcinoma	Grade III	2	0	0	Negative/Normal	Negative/Normal	Negative/Normal	No	N/A	058
TB15-086	P		P				52	Ductal carcinoma	Grade I:	1C	0	X	Positive/Elevated	Positive/Elevated	Negative/Normal	No	N/A	062
TB15-087	P		P				50	Ductal carcinoma	Grade II:	1C	0	X	Positive/Elevated	Positive/Elevated	Negative/Normal	No	N/A	063
TB15-088	P						56	Lobular carcinoma	Grade II:	3	3	1	Postive/elevated	Postive/elevated	HER-2 negative	Yes	N/A	41
TB15-089	P						79	Lobular carcinoma	Grade II:	2	1A	0	Postive/elevated	Postive/elevated	HER-2 negative	No	N/A	86
TB15-090	P						43	Lobular carcinoma	Grade II:	3	1A	X	Postive/elevated	Postive/elevated	HER-2 negative	No	N/A	80
TB15-091	P						58	Lobular carcinoma	Grade II:	2	2A	0	Postive/elevated	Postive/elevated	HER-2 negative	No	N/A	93
TB15-092	P		P				78	Lobular carcinoma	Grade II:	3	1A	X	Postive/elevated	Postive/elevated	HER-2 negative	No	N/A	79
TB15-093			P				51	Lobular carcinoma	Grade I:	1C	1A	0	Postive/elevated	Negative/normal	HER-2 negative	No	N/A	81
TB15-094	P		P				65	Lobular carcinoma	Grade II:	1C	2	0	Postive/elevated	Postive/elevated	FISH-negative	No	N/A	66
TB15-095	P		P				61	Lobular carcinoma	Grade I:	1B	1A	X	Postive/elevated	Postive/elevated	HER-2 negative	No	N/A	59
TB15-096			P				73	Lobular carcinoma	Grade II:	3	2		Postive/elevated	Postive/elevated	FISH - positive	No	N/A	12
TB15-097	P						45	Lobular carcinoma	Grade II:	3	1M		Postive/elevated	Postive/elevated	HER-2 negative	No	N/A	57
TB15-099			P				62	Lobular carcinoma	Grade II:	1C	0	0	Postive/elevated	Postive/elevated	HER-2 negative	No	N/A	89
TB15-100	P		P				69	Lobular carcinoma	Grade II:	1C	0	0	Postive/elevated	Postive/elevated	HER-2 positive	No	N/A	79
TB15-101			P	P			74	Lobular carcinoma	Grade II:	1C	0	X	Postive/elevated	Postive/elevated	HER-2 negative	No	N/A	80
TB15-102			P				69	Lobular carcinoma	Grade II:	1C	0	0	Postive/elevated	Postive/elevated	HER-2 negative	No	N/A	72
TB15-103			P				81	Lobular carcinoma	Grade III	2	0	0	Postive/elevated	Postive/elevated	HER-2 negative	No	N/A	75
TB15-104	P		P				70	Lobular carcinoma	Grade II:	2	0	0	Postive/elevated	Postive/elevated	HER-2 negative	Yes	68	70
TB15-105	P	P	P	P			47	Lobular carcinoma	Grade II:	1B	0	0	Postive/elevated	Postive/elevated	HER-2 negative	No	N/A	62
TB15-106	P	P	P				43	Lobular carcinoma	Grade II:	1C	0	0	Negative/normal	Negative/normal	HER-2 negative	No	N/A	65
TB15-107	P	P	P				83	Lobular carcinoma	Grade II:	2	0	X	Postive/elevated	Negative/normal	HER-2 negative	No	N/A	63
TB15-108			P				49	Lobular carcinoma	Grade II:	2	0	X	Postive/elevated	Negative/normal	HER-2 negative	No	N/A	68
TB15-109	P	P	P				48	Lobular carcinoma	Grade II:	1C	0	0	Postive/elevated	Postive/elevated	HER-2 negative	No	N/A	65
TB15-110			P				78	Lobular carcinoma	Grade II:	1C	0	0	Postive/elevated	Postive/elevated	HER-2 negative	Yes	N/A	73
TB15-111			P	P			51	Lobular carcinoma	Grade II:	1C	0	0	Postive/elevated	Postive/elevated	HER-2 negative	No	N/A	68
TB15-112	P	P	P				62	Lobular carcinoma	Grade II:	3	1A	0	Postive/elevated	Postive/elevated	HER-2 negative	No	N/A	66
TB15-113	P	P	P				59	Lobular carcinoma	Grade II:	2	0	X	Postive/elevated	Negative/normal	HER-2 negative	No	N/A	62
TB15-116	P		P				72	Lobular carcinoma	Grade I:	1C	0	0	Positive/Elevated	Positive/Elevated	Negative/Normal	No	N/A	061
TB15-117	P		P				46	Lobular carcinoma	Grade III	2	0	0	Negative/Normal	Negative/Normal	Negative/Normal	No	N/A	063
TB15-118	P		P				61	Lobular carcinoma	Grade II:	2	0	X	Positive/Elevated	Positive/Elevated	Negative/Normal	No	N/A	060
TB15-119			P				64	Lobular carcinoma	Grade II:	1C	1M	X	Postive/elevated	Postive/elevated	HER-2 negative	No	N/A	61
TB15-121	P		P				55	Lobular carcinoma	Grade II:	2	2A	0	Postive/elevated	Postive/elevated	HER-2 negative	No	N/A	95
TB15-122	P		P				49	Lobular carcinoma	Grade III	1B	2A	0	Postive/elevated	Postive/elevated	HER-2 negative	No	N/A	83
TB15-123	P		P				65	Lobular carcinoma	Grade II:				Postive/elevated	Postive/elevated	HER-2 positive	No	N/A	73
TB15-124	P		P				48	Lobular carcinoma	Grade II:	IS	0	0	Positive/Elevated	Positive/Elevated	Negative/Normal	No	N/A	103
		P=postive	Blank=negative															

Supplemental table 2 Colon Cancer														
**Case**	**TRMT11-GRIK2**	**MTOR-TP53BP1**	**CCNH-C50RF30**	**KDM4-AC011523.2**	**TMEM135-CCDC67**	**LRRC59-FLJ60017**	**Sex**	**Age**	**Grade**	**pT**	**pN**	**pM**	**Site of Distant Metastasis**	**Recur (months)**
K301							M	51	Grade II: Mod diff, mod well diff,	2	2	0	None	0
KB071							M	62	Grade II: Mod diff, mod well diff,	2	2	0	None	0
RS08-032							F	56	Grade II: Mod diff, mod well diff,	3	2	X	None	0
RS08-074	P						M	52	Grade III: Poorly differentiated, d	3	2	X	None	0
TB15-012			P				M	52	Grade I: Well differentiated, diffe	3	2	0	None	0
TB15-013							M	72	Grade II: Mod diff, mod well diff,	3	1	X	Lung	0
TB15-014							M	55	Grade II: Mod diff, mod well diff,	3	1	0	None	41
TB15-015							M	50	Grade II: Mod diff, mod well diff,	2	1	0	None	0
TB15-016	P		P				M	90	Grade II: Mod diff, mod well diff,	3	1	X	None	0
TB15-017							F	52	Grade II: Mod diff, mod well diff,	2	1	X	None	0
TB15-018			P				F	71	Grade III: Poorly differentiated, d	4	1	0	None	0
TB15-019							M	84	Grade II: Mod diff, mod well diff,	3	1	0	None	0
TB15-020			P				F	55	Grade II: Mod diff, mod well diff,	3	2	0	Lung	31
TB15-021	P		P				F	84	Grade II: Mod diff, mod well diff,	3	2	0	None	0
TB15-022	P		P				M	81	Grade II: Mod diff, mod well diff,	3	1	X	None	19
TB15-023							M	61	Grade II: Mod diff, mod well diff,	3	1	X	None	0
TB15-024							M	53	Grade II: Mod diff, mod well diff,	3	0	0	None	0
TB15-025							M	74	Grade II: Mod diff, mod well diff,	2	0	0	None	0
TB15-026							F	48	Grade II: Mod diff, mod well diff,	3	0	0	None	0
TB15-027			P				F	85	Grade II: Mod diff, mod well diff,	1	0	X	None	0
TB15-028			P				F	42	Grade II: Mod diff, mod well diff,	3	0	0	None	0
TB15-029			P				F	51	Grade II: Mod diff, mod well diff,	3	0	0	None	0
TB15-030							M	59	Grade II: Mod diff, mod well diff,	2	0	0	None	0
TB15-031							F	78	Grade II: Mod diff, mod well diff,	3	0	X	None	0
TB15-032							M	50	Grade II: Mod diff, mod well diff,	3	0	0	None	0
TB15-033			P				F	75	Grade II: Mod diff, mod well diff,	3	0	0	None	0
TB15-034			P				M	64	Grade II: Mod diff, mod well diff,	3	0	X	None	0
TB15-035							M	59	Grade II: Mod diff, mod well diff,	3	0	0	None	0
TB15-036							F	50	Grade II: Mod diff, mod well diff,	3	0	0	None	0
TB15-037	P						M	76	Grade II: Mod diff, mod well diff,	3	0	0	None	0
TB15-038	P		P				F	92	Grade III: Poorly differentiated, d	3	0	X	None	0
TB15-039							M	70	Grade II: Mod diff, mod well diff,	2	0	0	None	0
TB15-040							F	50	Grade II: Mod diff, mod well diff,	3	0	0	None	0
TB15-041							M	72	Grade II: Mod diff, mod well diff,	2	0	0	None	0
TB15-042	P						M	62	Grade II: Mod diff, mod well diff,	3	0	0	None	0
TB15-043							F	60	Grade II: Mod diff, mod well diff,	1	0	X	None	0
TB15-044			P				M	71	Grade III: Poorly differentiated, d	3	0	X	None	0
TB15-045	P						F	66	Grade I: Well differentiated, diffe	1	0	0	None	0
TB15-046	P						M	93	Grade III: Poorly differentiated, d	3	0	0	None	0
TB15-047	P		P				F	51	Grade II: Mod diff, mod well diff,	2	0	0	None	0
TB15-048	P		P				F	79	Grade II: Mod diff, mod well diff,	3	0	0	None	0
TB15-049							M	64	Grade II: Mod diff, mod well diff,	2	0	X	None	0
TB15-050	P	P					F	80	Grade II: Mod diff, mod well diff,	3	0	X	None	0
TB15-051	P		P				M	50	Grade II: Mod diff, mod well diff,	3	0	0	None	0
TB15-052	P						M	74	Grade II: Mod diff, mod well diff,	2	1	X	None	0
TB15-053	P		P				M	68	Grade II: Mod diff, mod well diff,	3	2	X	None	0
TB15-054	P	P	P				M	57	Grade II: Mod diff, mod well diff,	3	1	0	None	0
TB15-055	P	P	P				F	73	Grade II: Mod diff, mod well diff,	4	2	0	None	0
TB15-056	P	P	P				M	66	Grade III: Poorly differentiated, d	3	2	X	None	15
TB15-057	P		P				F	57	Grade II: Mod diff, mod well diff,	3	1	1	Liver	0
TB15-058	P						M	67	Grade III: Poorly differentiated, d	3	2	0	None	0
TB15-059	P		P				M	46	Grade II: Mod diff, mod well diff,	3	1	0	None	0
TB15-075	P						F	66	Grade II: Mod diff, mod well diff,	3	0	X	None	0
TB15-098	P		P					68	Grade II: Mod diff, mod well diff,	3	0	X	None	0
TP08-S00292			P				F	73	Grade II: Mod diff, mod well diff,	3	1	X	None	0
TP08-S00620	P						F	71	Grade II: Mod diff, mod well diff,	3	1	X	None	0
TP08-S00673			P				F	69	Grade II: Mod diff, mod well diff,	3	1	X	None	0
TP08-S00678							M	78	Grade II: Mod diff, mod well diff,	3	1	X	None	0
TP09-P317	P		P				F	82	Grade II: Mod diff, mod well diff,	2	1	0	None	0
TP09-S0551							M	63	Grade II: Mod diff, mod well diff,	2	1	X	None	0
		P=postive	Blank=negative											

Supplemental table 3 Esophageal Adenocarcinoma						
**Case**	**TRMT11-GRIK2**	**MTOR-TP53BP1**	**CCNH-C50RF30**	**KDM4-AC011523.2**	**TMEM135-CCDC67**	**LRRC59-FLJ60017**
994,995 T	P					
15995 TE			P			
16115 TE			P			
18787 TE	P		P			
41791 TE			P			
40320 TE	P		P			
40498 TE			P			
41081 TE			P			
15938 TE	P					
16806 TE	P					
18734 TE						
19061 TE			P			
43526 TE	P		P			
44636 TE						
43872 TE						
43888 TE			P			
41285 TE	P					
41701 TE	P					
41744 TE						
42709 TE			P			
44231 TE			P			
44014 TE						
44267 TE			P			
43075 TE	P					
40776 TE			P			
40371 TE			P			
19487 TE			P			
40449 TE			P			
40107 TE			P			
41032 TE			P			
11913 TE			P			
14020 TE			P			
11836 TE			P			
13516 TE			P			
		P=postive	Blank=negative			

Supplemental table 4 Hepatocellular Carcinoma

Case	TRMT11-GRIK2	MTOR-TP53BP1	CCNH-C5ORF30	KDM4-AC011523.2	TMEM135-CCDC67	LRRC59-FLJ60017	sex	Race	Age at Op.	Patient_ Status	PrimaryCauseofDeath	Recurrence	Patient Survival Days
HCC90							M	White	81	Dead	Unknown Cause	No	426
HCC120							M	White	51	Dead	Sepsis	No	998
HCC187							M	White	76	Dead		Yes	N/A
HCC'92							M	White	78	Dead	Graft Failure, Recurrent Disease	No	1577
HCC119			P				M	White	55	Alive		Yes	3894
HCC121							M	White	51	Dead	Malignancy, Specify	Yes	968
HCC163			P				F	White	68	Dead	Cardiac	No	3519
HCC162							M	White	86	Dead	Other, Specify	No	28
HCC112			P				F	White	44	Dead	Malignancy, Recurrent, Specify	Yes	492
HCC113			P				M	White	79	Dead	Infection, Fungal	No	43
HCC135							F	White	74	Dead	Cerebrovascular, Embolic Stroke	No	4037
HCC95							M	White	76	Dead	Malignancy, Recurrent, Specify	Yes	927
HCC94			P				M	White	88	Dead	Malignancy, Recurrent, Specify	Yes	900
HCC117			P				M	White	60	Dead	Malignancy, Recurrent, Specify	Yes	515
HCC140							M	White	85	Dead	Malignancy, Recurrent, Specify	Yes	740
HCC110			P				M	White	73	Dead	Malignancy, Recurrent, Specify	Yes	1802
HCC111							M	White	66	Dead	Malignancy, Recurrent, Specify	Yes	1115
HCC134							F	White	39	Dead	Malignancy, Recurrent, Specify	Yes	199
HCC136							M	White	89	Dead	Malignancy, Recurrent, Specify	Yes	173
HCC138			P				F	Black	70	Alive		No	7485
HCC114			P				M	White	76	Dead	Malignancy, Recurrent, Specify	Yes	194
HCC141			P				M	White	84	Dead	Malignancy, Specify	Yes	1396
HCC142							M	Black	41	Dead	Infection, Fungal	No	28
HCC137			P				M	White	61	Alive		Yes	9311
HCC139							M	White	83	Dead	Cardiovascular, Myocardial Infarct	No	878
HCC96			P				M	White	83	Dead	Infection, Bacterial Sepsis	No	28
HCC165							M	White	82	Dead	Malignancy, Recurrent, Specify	Yes	482
HCC164			P				M	White	87	Dead	Pulmonary Infection/Pneumonia	No	214
HCC109							F	White	59	Dead	Malignancy, Recurrent, Specify	Yes	756
HCC159							M	White	76	Dead	Malignancy, Recurrent, Specify	Yes	1431
HCC160	P		P				M	White	78	Dead	Malignancy, Specify	Yes	139
HCC108							M	White	75	Dead	Malignancy, Recurrent, Specify	Yes	1416
HCC152	P						F	White	75	Dead	Malignancy, Recurrent, Specify	Yes	276
HCC115			P				M	White	66	Alive		No	8783
HCC146			P				M	White	85	Dead	Pulmonary Infection/Pneumonia	No	45
HCC156							M	White	70	Dead	Graft Failure, Recurrent Disease	No	489
HCC157	P						M	White	84	Dead	Unknown Cause	No	2954
HCC151	P		P				M	White	80	Dead	Malignancy, Recurrent, Specify	Yes	265
HCC149							F	White	67	Dead	Malignancy, Recurrent, Specify	Yes	1014
HCC148							M	White	82	Dead	Infection, Fungal	No	19
HCC81	P						F	White	81	Alive		No	6737
HCC154							M	White	78	Dead	Malignancy, Recurrent, Specify	Yes	394
HCC178			P				M	White	61	Dead	Malignancy, Recurrent, Specify	Yes	856
HCC153							M	White	83	Dead	Liver Dis, Ascending Cholangitis	No	176
HCC169			P				M	White	71	Dead	Malignancy, Specify	Yes	1776
HCC88			P				M	White	82	Alive		No	6326
HCC89			P				F	White	63	Dead	Graft Failure, Infection	No	227
HCC168			P				M	White	81	Dead	Malignancy, Recurrent, Specify	Yes	381
HCC85			P				M	White	56	Dead	Malignancy, Recurrent, Specify	Yes	638
HCC173							M	Asian	71	Dead	Malignancy, Recurrent, Specify	Yes	2435
HCC87							M	White	69	Dead	Unknown Cause	No	174
HCC86			P				F	White	77	Dead	Cardiovascular, Cardiac Arrest	No	3376
HCC182			P				M	White	64	Dead	Bile Duct Problem	No	40
HCC179	P						F	White	84	Dead	Cardiovascular, Other, Specify	No	3019
HCC99			P				M	White	77	Dead	Infection, Fungal	No	61
HCC98			P				M	White	78	Dead	Cardiovascular, Myocardial Infarct	No	3190
HCC102	P		P				M	White	67	Alive		No	4960
HCC97			P				M	White	74	Dead	Malignancy, Recurrent, Specify	Yes	1695
HCC177							M	White	70	Dead	Multiple Organ Failure	No	3835
HCC02			P				M	Asian	63	Alive		No	1500
HCC184							M	White	66	Dead	Malignancy, Recurrent, Specify	Yes	678
HCC7							M	White	67	Dead		Yes	N/A
HCC27							M	White	56	Dead		Yes	N/A
HCC06			P				M	White	74	Alive		No	1473
HCC12							M	White	66	Dead		Yes	N/A
HCC10							M	White	78	Dead		Yes	N/A
HCC23			P				F	White	67	Alive		No	1317
HCC17	P						M	White	57	Alive		No	1365
HCC28	P		P				M	White	56	Dead		Yes	N/A
HCC71							M	White	51	Alive		No	792
		P=postive	Blank=negative										

Supplemental table 5 Non-Small Cell Lung Cancer

Case	TRMT11-GRIK2	MTOR-TP53BP1	CCNH-C50RF30	KDM4-AC011523.2	TMEM135-CCDC67	LRRC59-FLJ60017	age	Sex	Race	pT	pN	pM	Histology	5-year survival
1L	P						78	Female	White	2	0	0	Squamous cell carcinoma, NOS	Alive
2L	P		P				80	Male	White	2	0	0	Squamous cell carcinoma, NOS	Deceased
3L	P		P				58	Female	Black	2	0	0	Squamous cell carcinoma, keratinizi	Deceased
4L		P	P				74	Male	White	2	0	0	Squamous cell carcinoma, NOS	Deceased
5L			P				70	Female	White	2	2	0	Squamous cell carcinoma, NOS	Deceased
6L							76	Female	White	1	0	0	Squamous cell carcinoma, NOS	Deceased
7L							74	Female	Black	3	0	0	Squamous cell carcinoma, clear cell	Deceased
8L			P				75	N/A	N/A	2	0	0	Squamous cell carcinoma, NOS	Alive
9L	P						80	Male	White	3	0	0	Squamous cell carcinoma, NOS	Deceased
10L	P		P				71	Female	White	2	0	0	Squamous cell carcinoma, NOS	Alive
11L	P	P					82	Female	White	1	0	0	Squamous cell carcinoma, NOS	Deceased
12L	P						75	Male	White	2	0	0	Squamous cell carcinoma, NOS	Deceased
13L	P						81	Male	White	2	0	0	Squamous cell carcinoma, NOS	Deceased
14L	P						80	Male	White	2	1	0	Squamous cell carcinoma, NOS	Deceased
15L	P						72	Male	White	2	1	0	Squamous cell carcinoma, NOS	Deceased
16L							52	Male	White	2	0	0	Squamous cell carcinoma, NOS	Deceased
17L							71	Female	White	2	0	0	Squamous cell carcinoma, NOS	Deceased
18L							71	Male	White	2	1	0	Squamous cell carcinoma, NOS	Deceased
19L	P						71	Female	White	2	0	0	Squamous cell carcinoma, keratinizi	Deceased
20L	P		P				65	Male	Black	1	2	0	Squamous cell carcinoma, NOS	Alive
21L	P		P		P		69	Male	White	2	0	0	Squamous cell carcinoma, NOS	Deceased
22L	P						73	Male	White	2	1	0	Squamous cell carcinoma, NOS	Deceased
23L							75	Female	White	2	0	0	Squamous cell carcinoma, NOS	Deceased
24L							71	Male	White	2	1	0	Squamous cell carcinoma, NOS	Alive
25L							74	Male	White	2	1	0	Squamous cell carcinoma, NOS	Alive
26L							77	N/A	N/A	2	0	0	Squamous cell carcinoma, NOS	Alive
27L							67	Male	White	2	0	0	Squamous cell carcinoma, NOS	Deceased
28L	P	P	P				62	Female	White	2	0	0	Squamous cell carcinoma, NOS	Alive
29L			P				82	Male	White	2	1	0	Squamous cell carcinoma, NOS	Deceased
30L			P				67	Male	White	2	1	0	Squamous cell carcinoma, NOS	Deceased
31L	P		P				74	Male	White	2	0	0	Squamous cell carcinoma, NOS	Deceased
32L							59	Male	White	2	0	1	Squamous cell carcinoma, NOS	Deceased
33L			P				83	Male	White	2	0	0	Squamous cell carcinoma, NOS	Deceased
34L			P				64	Male	White	2	0	0	Squamous cell carcinoma, lg cell, no	Deceased
35L	P		P				68	Female	White	2	0	0	Squamous cell carcinoma, NOS	Deceased
36L			P				73	Male	White	2	1	0	Squamous cell carcinoma, NOS	Deceased
37L			P				73	Male	White	2	1	0	Squamous cell carcinoma, NOS	Deceased
38L	P	P	P				76	Female	White	1	0	0	Squamous cell carcinoma, NOS	Deceased
39L	P						60	Female	White	2	1	0	Squamous cell carcinoma, NOS	Deceased
40L	P		P				56	Female	White	1	2	0	Squamous cell carcinoma, NOS	Deceased
41L							56	Male	Black	2	2	0	Squamous cell carcinoma, keratinizi	Deceased
42L	P					P	74	Female	White	2	0	0	Squamous cell carcinoma, NOS	Deceased
43L	P						77	Female	White	1	0	0	Squamous cell carcinoma, NOS	Deceased
44L						P	49	Female		2	0	0	Squamous cell carcinoma, NOS	Deceased
45L						P	73	Female	White	2	1	0	Squamous cell carcinoma, NOS	Deceased
46L	P					P	65	Female	White	2	1	0	Squamous cell carcinoma, NOS	Alive
47L			P			P	84	Male	White	1	0	0	Squamous cell carcinoma, NOS	Deceased
48L	P						78	Male	White	2	1	0	Squamous cell carcinoma, NOS	Deceased
49L			P			P	69	Female	White	4	0	0	Squamous cell carcinoma, NOS	Deceased
50L	P					P	71	Female	White	1	0	0	Squamous cell carcinoma, NOS	Alive
51L	P						79	Female	White	2	0	0	Squamous cell carcinoma, NOS	Alive
52L	P						61	Female	Black	1	0	0	Squamous cell carcinoma, NOS	Deceased
53L	P						56	Male	White	4	0		Squamous cell carcinoma, NOS	Deceased
54L							63	Female	Black	1	0	0	Squamous cell carcinoma, NOS	Deceased
55L						P	61	Female	White	1	0	0	Squamous cell carcinoma, NOS	Alive
56L							60	Female	White	4	1	0	Squamous cell carcinoma, keratinizi	Deceased
57L							76	Male	White	4	0	0	Squamous cell carcinoma, NOS	Deceased
58L	P						65	Male	White	3	2	0	Squamous cell carcinoma, keratinizi	Deceased
59L							68	Female	White	2	0	0	Squamous cell carcinoma, NOS	Alive
60L							76	Male	White	4	1	0	Squamous cell carcinoma, NOS	Deceased
61L							75	Male	Black	2	0	0	Squamous cell carcinoma, NOS	Alive
62L	P	P	P				68	Male	White	1	0	0	Squamous cell carcinoma, NOS	Alive
63L							75	Female	Black	1	0	0	Squamous cell carcinoma, NOS	Deceased
64L	P		P				66	Male	White	1	2	0	Squamous cell carcinoma, NOS	Deceased
65L			P			P	67	Male	White	2	0	0	Squamous cell carcinoma, NOS	Alive
66L			P				84	Male	White	2	1	0	Squamous cell carcinoma, NOS	Deceased
67L			P			P	55	Female	White	4	1	0	Squamous cell carcinoma, NOS	Alive
68L							66	Female	White	2	1	0	Squamous cell carcinoma, NOS	Alive
69L			P				84	Male	White	2	0	0	Squamous cell carcinoma, NOS	Deceased
70L			P				53	F	White	1	2	0	Squamous cell carcinoma, NOS	Alive
71L							66	F	White	1	0	0	Squamous cell carcinoma, NOS	Deceased
72L							57	F	White	2	0	0	Squamous cell carcinoma, NOS	Alive
73L			P				63	N/A	N/A	2	0	0	Squamous cell carcinoma, NOS	Alive
74L							64	M	Pacif	4	2	0	Squamous cell carcinoma, NOS	Deceased
75L							52	M	White	2	0	0	Squamous cell carcinoma, NOS	Alive
76L		P					74	M	White	2	2	0	Squamous cell carcinoma, NOS	Deceased
77L							44	F	White	1	2	0	Squamous cell carcinoma, NOS	Deceased
78L							57	M	White	3	0	0	Squamous cell carcinoma, NOS	Deceased
79L							80	M	White	2	1	0	Squamous cell carcinoma, NOS	Alive
80L							60	M	White	1	1	0	Squamous cell carcinoma, NOS	Deceased
81L							74	M	White	2	0	0	Squamous cell carcinoma, NOS	Alive
82L							65	F	White	1	1	0	Squamous cell carcinoma, NOS	Alive
83L			P				44	M	Black	1	0	0	Squamous cell carcinoma, NOS	Alive
84L							82	F	White	2	2	0	Squamous cell carcinoma, NOS	Deceased
85L							56	F	Black	2	0	0	Squamous cell carcinoma, NOS	Alive
86L		P	P			P	76	M	White	2	2	0	Squamous cell carcinoma, NOS	Deceased
87L							63	M	White	2	0	0	Squamous cell carcinoma, NOS	Alive
88L							57	M	White	2	1	0	Squamous cell carcinoma, NOS	Alive
89L	P						67	M	White	1	0	0	Squamous cell carcinoma, NOS	Alive
90L							41	F	White	2	0	0	Squamous cell carcinoma, NOS	Alive
91L							51	F	White	4	0	0	Squamous cell carcinoma, NOS	Alive
92L							75	M	Black	2	1	0	Squamous cell carcinoma, NOS	Deceased
93L						P	69	F	White	2	1	0	Squamous cell carcinoma, NOS	Alive
94L							68	F	White	1	0	0	Squamous cell carcinoma, NOS	Deceased
95L							65	F	White	2	1	0	Squamous cell carcinoma, NOS	Deceased
96L							58	M	White	1	0	0	Squamous cell carcinoma, NOS	Alive
97L							65	M	White	3	1	1	Squamous cell carcinoma, NOS	Deceased
98L							60	F	White	2	0	0	Squamous cell carcinoma, NOS	Deceased
99L							50	F	White	4	0	0	Squamous cell carcinoma, NOS	Deceased
100L							65	M	White	1	0	0	Squamous cell carcinoma, NOS	Deceased
101L	P						75	M	White	4	0	0	Squamous cell carcinoma, NOS	Deceased
		P=postive	Blank=negative											

Supplemental table 6 Ovarian Cancer

Case	TRMT11-GRIK2	MTOR-TP53BP1	CCNH-C5ORF30	KDM4-AC011523.2	TMEM135-CCDC67	LRRC59-FLJ60017	Age	Grade/Differentiation-Desc	pT	pN	pM	Month to Recur	Surv (Months)	Vital Statu
TB15-127	P						90	Grade I: Well differentiated, diffe	1A	0	0	0	78	Dead
TB15-128			P				59	Grade IV: Undifferentiated, anaplas	3B	0	0	0	80	Alive
TB15-129	P		P				41	Grade IV: Undifferentiated, anaplas	1	0	0	0	81	Alive
TB15-130	P		P				83	Grade I: Well differentiated, diffe	2A	0	0	0	85	Alive
TB15-131	P		P			P	61	Grade III: Poorly differentiated, d	1C	0	0	0	85	Alive
TB15-132	P						70	Grade III: Poorly differentiated, d	2C	0	0	0	76	Alive
TB15-133	P	P					44	Grade I: Well differentiated, diffe	1C	0	X	0	80	Alive
TB15-134							42	Grade III: Poorly differentiated, d	2A	0	X	0	64	Alive
TB15-135							61	Grade II: Mod diff, mod well diff,	1C	0	0	0	87	Alive
TB15-136							57	Grade II: Mod diff, mod well diff,	1A	0	0	0	60	Alive
TB15-137			P				52	Grade III: Poorly differentiated, d	1C	0	0	51	84	Alive
TB15-138							35	Grade IV: Undifferentiated, anaplas	1C	0	0	0	83	Alive
TB15-139	P						55	Grade I: Well differentiated, diffe	3B	0	X	0	86	Alive
TB15-140							56	Grade I: Well differentiated, diffe	1C	0	X	0	84	Alive
TB15-141	P					P	42	Grade III: Poorly differentiated, d	1C	0	0	0	58	Alive
TB15-142	P						44	Grade II: Mod diff, mod well diff,	1B	0	X	0	82	Alive
TB15-143	P						58	Grade III: Poorly differentiated, d	1C	0	0	0	80	Alive
TB15-144			P				50	Grade IV: Undifferentiated, anaplas	1A	0	0	0	66	Alive
TB15-145			P				45	Grade IV: Undifferentiated, anaplas	1C	0	X	0	73	Alive
TB15-146			P				49	Grade III: Poorly differentiated, d	1C	0	0	0	70	Alive
TB15-147			P				55	Grade III: Poorly differentiated, d	3B	0	0	0	64	Alive
TB15-148	P						44	Grade III: Poorly differentiated, d	1C	0	X	0	63	Alive
TB15-149			P				69	Grade II: Mod diff, mod well diff,	3B	0	X	0	62	Alive
TB15-150							63	Grade III: Poorly differentiated, d	3C	0	X	0	75	Dead
TB15-151	P		P				42	Grade II: Mod diff, mod well diff,	1A	0	0	0	93	Alive
TB15-152							50	Grade III: Poorly differentiated, d	1A	0	0	0	90	Alive
TB15-153	P		P				75	Grade I: Well differentiated, diffe	1A	0	0	0	91	Alive
TB15-154							59	Grade III: Poorly differentiated, d	2A	0	0	0	84	Alive
TB15-155							43	Grade I: Well differentiated, diffe	1A	0	0	0	89	Alive
TB15-156							42	Grade II: Mod diff, mod well diff,	1C	0	0	0	84	Alive
TB15-157	P		P				50	Grade IV: Undifferentiated, anaplas	1C	0	0	0	85	Alive
TB15-180	P	P				P	57	Grade IV: Undifferentiated, anaplas	3C	1	0	0	7	Dead
TB15-181	P	P					56	Grade III: Poorly differentiated, d	3C	1	X	0	24	Dead
TB15-182			P				68	Grade IV: Undifferentiated, anaplas	3C	1	0	10	39	Dead
TB15-183	P		P				81	Grade III: Poorly differentiated, d	3C	1	0	0	7	Dead
TB15-184			P				51	Grade III: Poorly differentiated, d	3	1	1	16	75	Dead
TB15-185			P				51	Grade II: Mod diff, mod well diff,	3C	1	1	0	80	Alive
TB15-186	P		P				57	Grade III: Poorly differentiated, d	3C	1	1	0	9	Dead
TB15-187	P		P				41	Grade III: Poorly differentiated, d	3C	1	0	0	74	Alive
TB15-188			P				70	Grade III: Poorly differentiated, d	3C	1	1	0	58	Dead
TB15-189	P		P				66	Grade IV: Undifferentiated, anaplas	3C	1	0	36	57	Dead
TB15-190			P				65	Grade III: Poorly differentiated, d	3C	1	0	0	78	Alive
TB15-191							61	Grade III: Poorly differentiated, d	3C	1	1	19	34	Dead
TB15-192			P				52	Grade IV: Undifferentiated, anaplas	3C	1	1	0	3	Dead
TB15-193	P		P				70	Grade III: Poorly differentiated, d	3C	1	0	18	38	Dead
TB15-194	P		P				50	Grade IV: Undifferentiated, anaplas	3C	1	0	0	76	Alive
TB15-195							69	Grade III: Poorly differentiated, d	3C	1	0	8	9	Dead
TB15-196	P		P				57	Grade III: Poorly differentiated, d	3C	1	0	8	70	Alive
TB15-197	P		P				61	Grade III: Poorly differentiated, d	3C	1	0	47	58	Dead
TB15-198	P	P	P				48	Grade III: Poorly differentiated, d	3C	1	0	0	16	Dead
TB15-199							78	Grade III: Poorly differentiated, d	3C	1	1	9	30	Dead
TB15-207			P				64	Grade II: Mod diff, mod well diff,	3C	1	1	0	10	Dead
TB15-208			P				52	Grade III: Poorly differentiated, d	3C	1	1	0	2	Dead
TB15-209							66	Grade III: Poorly differentiated, d	3C	1	0	34	81	Dead
TB15-210	P						71	Grade III: Poorly differentiated, d	3C	1	0	0	30	Dead
TB15-211							78	Grade III: Poorly differentiated, d	3C	1	1	0	27	Dead
TB15-212			P				79	Grade III: Poorly differentiated, d	3C	1	1	0	78	Dead
TB15-213	P					P	77	Grade III: Poorly differentiated, d	3C	1	1	0	25	Dead
TB15-214							72	Grade III: Poorly differentiated, d	3C	1	0	6	11	Dead
TB15-215	P						68	Grade III: Poorly differentiated, d	3C	1	1	0	32	Dead
TB15-216							69	Grade IV: Undifferentiated, anaplas	3C	1	0	3	85	Dead
		P=postive	Blank=negative											

Supplemental table 7 Glioblastoma Multiforme

Cases	TRMT11-GRIK2	MTOR-TP53BP1	CCNH-C50RF30	KDM4-AC011523.2	TMEM135-CCDC67	LRRC59-FLJ60017	area	dx	sex	age at dx	date of de	survival days
GBM268			P				x	gbm	f	59.30411	42384	619
GBM269							fr, l	gbm	f	74.08219	42177	410
GBM270							fr, l	gbm	m	57.97534	42552	784
GBM284			P				par, r	gbm	f	72.99726	42058	290
GBM288			P				fr, l	gbm	f	72.07671	41992	192
GBM293							par, r	gbm	f	74.63288	41880	80
GBM301							x	gbm	m	87.73973	42200	383
GBM303			P				fr, r	gbm	f	43.22466	39592	250
GBM304			P				occ, l	gbm	m	42.72877	38631	261
GBM306			P				temp, r	gbm	m	56.28493	39639	588
GBM310							fr, l	gbm	m	47.24932	40152	642
GBM226			P			P	temp, l	gbm	m	60.32603	42552	1267
GBM227							x	gbm	m	74.30685	41367	29
GBM241			P				temp, l	gbm	m	60.86027	42552	873
GBM242			P				x	gbm	f	79.13151	42000	321
GBM245			P				temp, r	gbm	f	54.4411	42335	657
GBM212			P				x	gbm	m	49.85205	41518	768
GBM219							par, l	gbm	f	60.80274	41620	344
GBM220						P	temp, l	gbm	m	61.18082	41615	333
GBM231			P				front, l	gbm	m	89.71507	41678	32
GBM250			P				fr, r	gbm	f	75.58356	41890	201
GBM251			P				x	gbm	f	43.46027	42552	856
GBM252			P			P	x	gbm	m	59.30959	41711	10
GBM253			P				fr, r	gbm	m	56.84932	42552	848
GBM233			P				temp, l	gbm	m	80.50959	41733	87
GBM320							temp-par,	gbm	m	45.39726	39057	677
GBM323							temp, l	gbm	m	42.63562	42358	690
GBM236			P			P	par, r	gbm	f	82.38356	41697	37
GBM239			P				par, l	gbm	f	58.62466	41816	146
GBM137							x	gbm	f	74.37534	39241	205
GBM138							x	gbm	f	72.97808	39112	75
GBM139							x	gbm	f	25.93973	42552	3483
GBM142			P				x	gbm	f	82.20274	39269	190
GBM143						P	x	gbm	m	72.67671	39140	36
GBM146			P				x	gbm	m	68.38904	39638	532
GBM147			P				x	gbm	m	72.6274	39332	220
GBM150							x	gbm	f	75.72055	39547	428
GBM154			P				x	gbm	f	32.87123	39477	357
GBM159							x	gbm	m	60.43014	39189	69
GBM257							fr, r	gbm	m	66.87671	41788	72
GBM259							fr, l	gbm	m	56.83562	42331	607
GBM260			P				x	gbm	f	72.16438	42208	482
GBM160	P		P				x	gbm	f	63.53151	39352	161
GBM263	P		P				par-occ, r	gbm	f	50.42466	42552	821
GBM264							occ, l	gbm	f	81.90137	41853	122
GBM265							x	gbm	f	64.53151	42552	804
GBM267							x	gbm	m	58.28767	42552	791
GBM330			P				temp, r	gbm	f	53.13699	40024	1106
GBM331							par, r	gbm	f	44.57534	41335	2805
GBM332	P					P	temp, l	gbm	m	80.50959	41733	87
GBM165							x	gbm	f	69.4274	39466	257
GBM166							x	gbm	m	60.32603	39728	517
GBM167			P			P	x	gbm	m	45.72055	39498	257
GBM168							x	gbm	m	61.04658	39429	167
GBM170							x	gbm	f	58.25753	39550	262
GBM333			P				par, r	gbm	f	82.38356	41697	135
GBM99							temp, l	gbm	m	65.86849	38747	108
GBM104							temp, l	gbm	m	64.83014	40257	1550
GBM105							fr, l	gbm	f	62.4411	38925	188
GBM122			P				x	gbm	m	56.77808	39076	116
GBM123							x	gbm	m	75.72055	39033	73
GBM124		P	P				x	gbm	m	55.65479	39041	74
GBM103	P						temp, l	gbm	f	75.66027	39004	302
GBM106							temp, l	gbm	m	44.36438	39179	442
GBM96						P	x	gbm	f	78.3589	42552	3937
GBM95	P						x	gbm	m	33.4411	38922	349
GBM125						P	x	gbm	m	55.70411	39139	143
GBM117			P				x	gbm	m	52.15342	38978	96
GBM115							x	gbm	m	44.4274	39313	514
GBM135	P		P				x	gbm	f	38.23014	39735	713
GBM132	P	P	P				x	gbm	m	48.61096	39180	166
GBM131			P				x	gbm	f	81.33699	39031	18
GBM127							x	gbm	f	75.72055	39352	346
GBM119			P				x	gbm	f	56.73151	39377	436
GBM128			P				x	gbm	f	43.70137	39214	208
GBM136	P		P				x	gbm	m	78.61644	39304	281
GBM102							x	gbm	f	60.40548	39363	675
GBM100			P				par, l	gbm	f	69.53973	41122	2443
GBM98							x	gbm	m	58.12329	38798	173
GBM71							temp, l	gbm	m	53.35616	40680	2303
GBM80							x	gbm	m	67.80548	38475	67
GBM66			P				tem-par, l	gbm	m	64.46301	39278	1003
GBM67							x	gbm	m	57.19726	38445	152
GBM62			P				x	gbm	m	43.35616	38501	236
GBM54							temp, l	gbm	m	69.16986	38494	348
GBM53							temp, r	gbm	f	61.97534	38493	373
GBM65			P				fr, l	gbm	m	70.54247	38306	39
GBM86			P				fr, l	gbm	m	73.86027	38977	447
GBM82			P				x	gbm	f	46.44384	38543	103
GBM79			P				temp, r	gbm	m	50.99178	38830	436
GBM78							par, r	gbm	f	75.75616	39057	679
GBM83			P				par, r	gbm	m	37.30411	39448	1007
GBM84							fr, l	gbm	f	69.83288	38668	205
GBM87		P	P				par-occ, r	gbm	f	78.1863	38898	344
GBM17							x	gbm	f	77.08493	42552	2977
GBM18			P				x	gbm	f	65.21096	42157	398
GBM21	P		P				x	gbm	f	63.72877	40012	458
GBM24		P	P				x	gbm	m	56.19726	39896	315
GBM22	P		P				x	gbm	m	78.35616	39626	71
GBM27			P				x	gbm	f	85.03288	39943	360
GBM19			P				x	gbm	f	69.95616	39773	660
GBM13		P	P				temp, r	gbm	f	53.13699	40024	1106
GBM12		P	P				temp, l	gbm	m	42.63562	42358	690
GBM10			P				fr, l	gbm	m	47.24932	40152	642
GBM11			P				temp-par,	gbm	m	45.39726	39057	677
GBM15			P				par, r	gbm	f	44.57534	41335	2805
GBM2		P	P				fr, r	gbm	f	43.22466	39592	250
GBM7			P				temp, r	gbm	m	56.28493	39639	588
GBM5							occ, l	gbm	m	42.72877	38631	261
GBM46			P				x	gbm	m	65.76164	38057	113
GBM29	P		P				x	gbm	f	60.35068	42552	2914
GBM50			P				x	gbm	m	42.80822	38041	96
GBM32	P		P				x	gbm	m	63.48767	42131	485
GBM39	P		P				temp, r	gbm	m	55.30685	38280	11
GBM40			P				occ, r	gbm	f	69.27945	38300	362
GBM60			P				x	gbm	m	48.31781	38493	316
GBM177	P						x	gbm	m	65.11507	40198	700
GBM178			P				x	gbm	m	80.86575	39659	149
GBM176	P						x	gbm	f	55.89863	39925	495
GBM184							x	gbm	m	61.83562	41396	1793
GBM185							x	gbm	m	58.80822	40433	830
GBM186	P					P	x	gbm	f	60.43288	40126	516
GBM187					P		x	gbm	m	71.24384	39806	142
GBM188						P	x	gbm	m	65.62466	39997	329
GBM189					P	P	x	gbm	f	56.66301	40510	835
GBM198	P		P				x	gbm	f	61.06575	42049	2054
GBM190	P		P			P	x	gbm	m	55.95342	40354	669
GBM191	P		P				x	gbm	m	84.60274	39984	276
GBM192	P						x	gbm	f	71.88493	40088	365
GBM193						P	temp, r	gbm	m	28.32329	41940	2163
GBM194							x	gbm	m	73.0411	40015	237
GBM195	P		P			P	x	gbm	f	73.74247	39838	58
GBM196	P					P	x	gbm	m	80.53425	40007	180
GBM197							x	gbm	m	84.04384	40008	35
GBM199			P				x	gbm	m	72.20822	40087	23
GBM200			P				x	gbm	m	51.73425	42552	2095
GBM201	P		P				x	gbm	m	58.34521	42552	2025
GBM202			P				x	gbm	m	44.24658	40831	221
GBM203			P				x	gbm	f	53.74795	41097	459
GBM204							x	gbm	m	75.04658	40722	61
GBM205	P		P				x	gbm	f	38.71507	41336	625
GBM179							x	gbm	m	54.41096	39631	93
GBM180							x	gbm	m	61.66301	40372	820
GBM181							x	gbm	f	60.57534	39565	15
GBM183			P				x	gbm	f	51.92055	40541	940
GBM182	P						x	gbm	f	44.08219	42552	2991
GBM173							x	gbm	f	62.86575	39394	91
GBM172							x	gbm	m	77.70685	39498	202
GBM175			P				x	gbm	m	58.03836	39650	286
GBM174	P						x	gbm	f	82.0411	39925	575
		P=postive	Blank=negative									

Supplemental figure 1: Sanger sequencing representation of CCNH-C5orf30. (A) Sanger sequencing of CCNH-C5orf30 fusion transcript from cell lines. (B) Sanger sequencing of CCNH-C5orf30 fusion transcript from clinical tumor specimens.

Supplemental figure 2: Sanger sequencing representation of TRMT11-GRIK2. (A) Sanger sequencing of TRMT11-GRIK2 fusion transcript from cell lines. (B) Sanger sequencing of TRMT11-GRIK2 fusion transcript from clinical tumor specimens.

Supplemental figure 3: Sanger sequencing representation of LRRC59-FLJ60017. (A) Sanger sequencing of LRRC59-FLJ60017 fusion transcript from cell lines. (B) Sanger sequencing of LRRC59-FLJ60017 fusion transcript from clinical tumor specimens.

Supplemental figure 4: Sanger sequencing representation of TMEM135-CCDC67 from clinical tumor specimens.

Supplemental figure 5: Sanger sequencing representation of mTOR-TP53BP1 from clinical tumor specimens.

Supplemental figure 6: Sanger sequencing representation of KDM4-AC011523.2 from clinical tumor specimens.

Supplemental figure 1A. CCNH-C5orf30 fusion

Cell lines

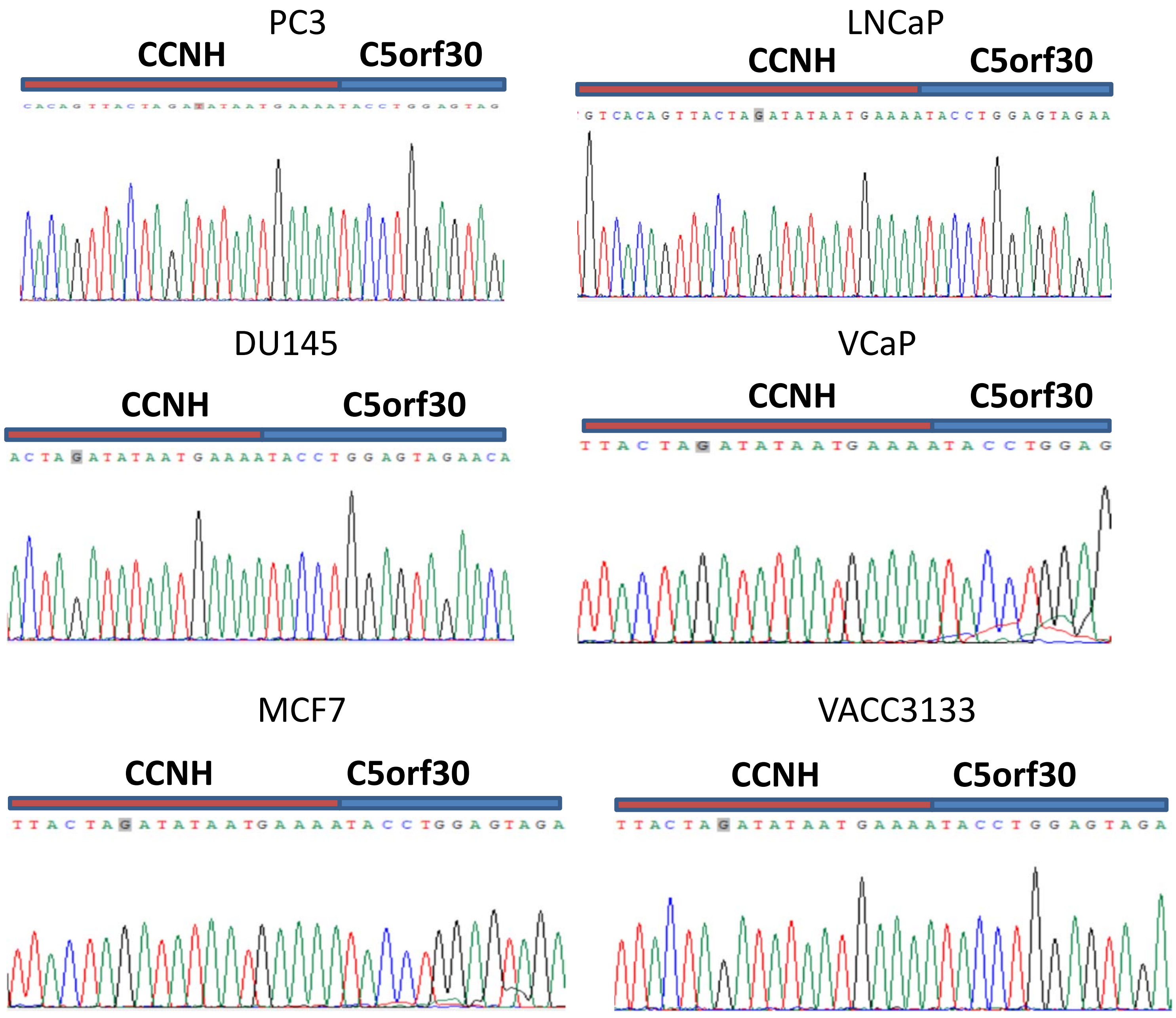

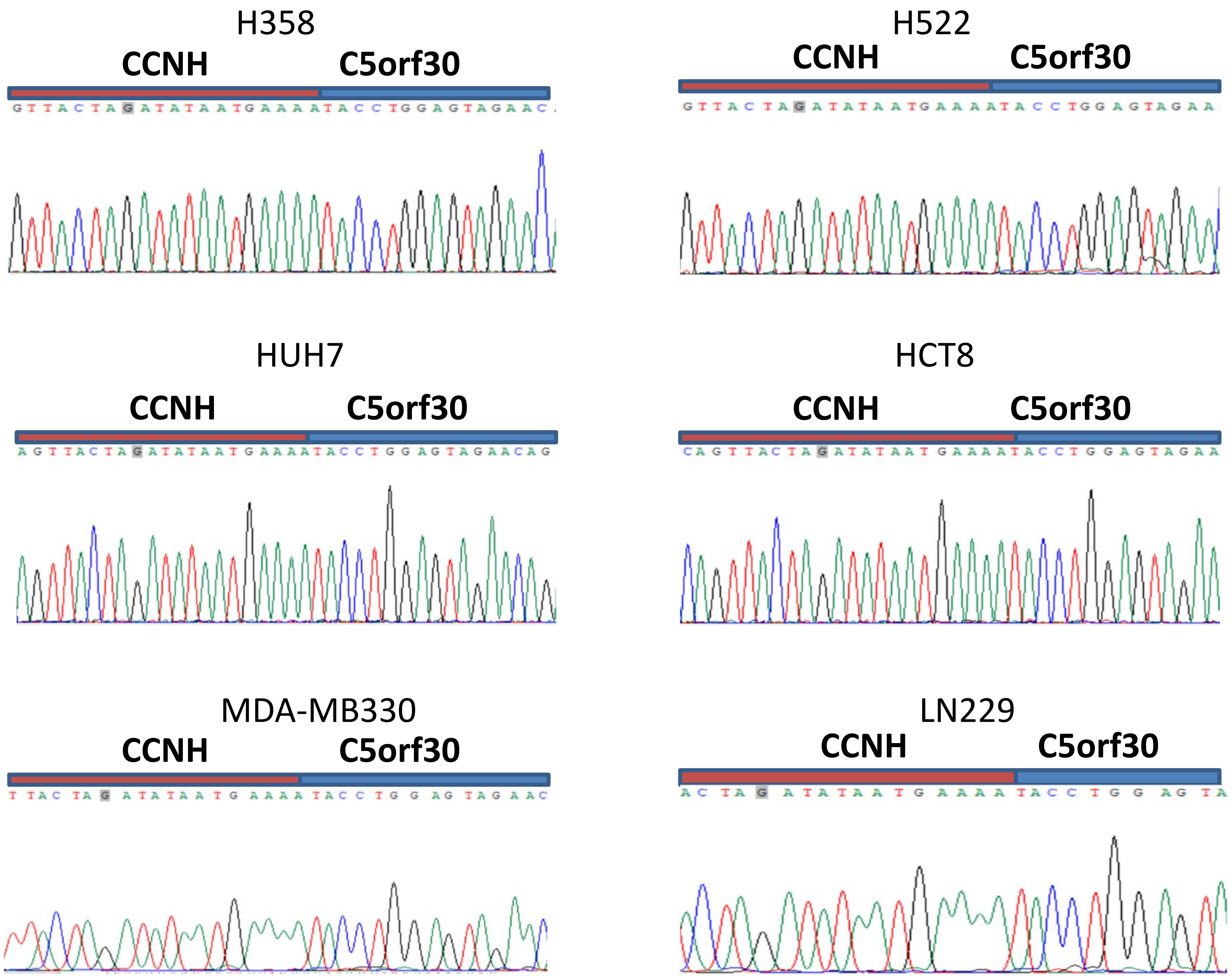
H358
CCNH
C5orf30
H522
CCNH
C5orf30
HUH7
CCNH
C5orf30
HCT8
CCNH
C5orf30
MDA-MB330
CCNH
C5orf30
LN229
CCNH
C5orf30

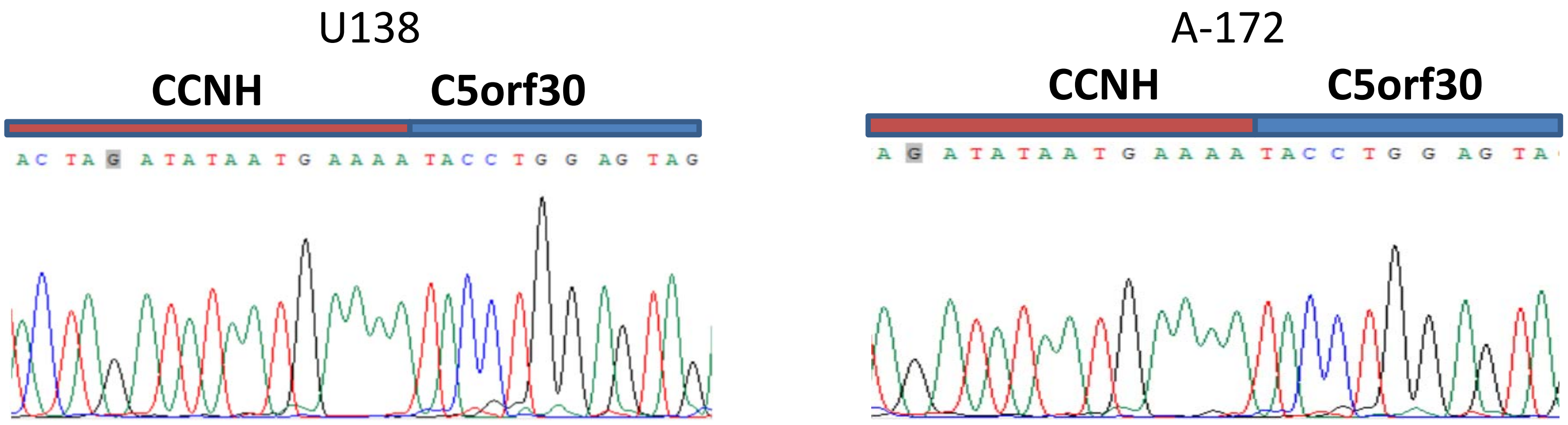
U138
CCNH
C5orf30
AC TA G ATATAATG AAAA TACC TG G AG TAG
A-172
CCNH
C5orf30
A G ATATAATG AAAA TACC TG G AG TA

Supplemental figure 1B.

CCNH-C5orf30 fusion

Clinical specimens

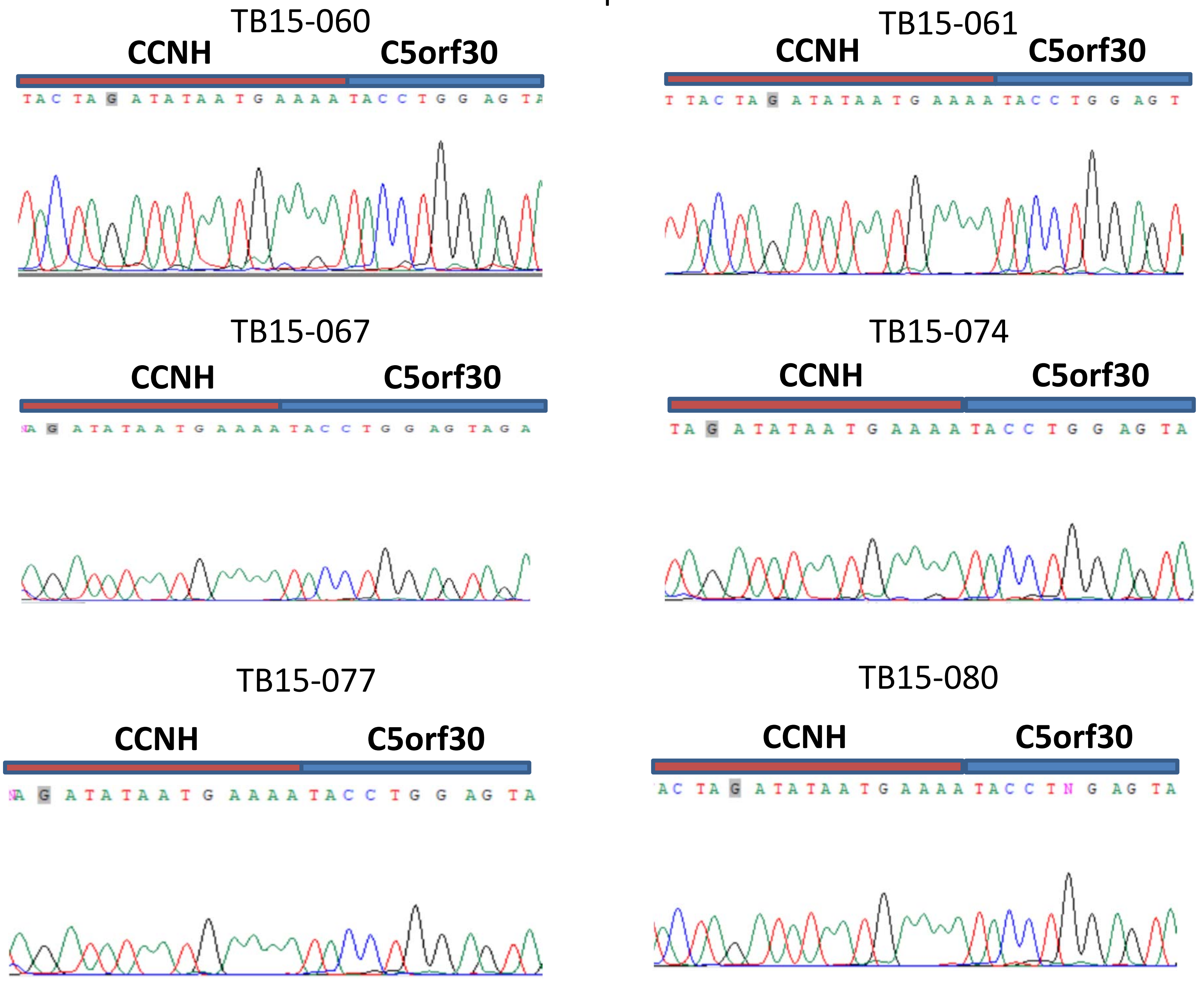

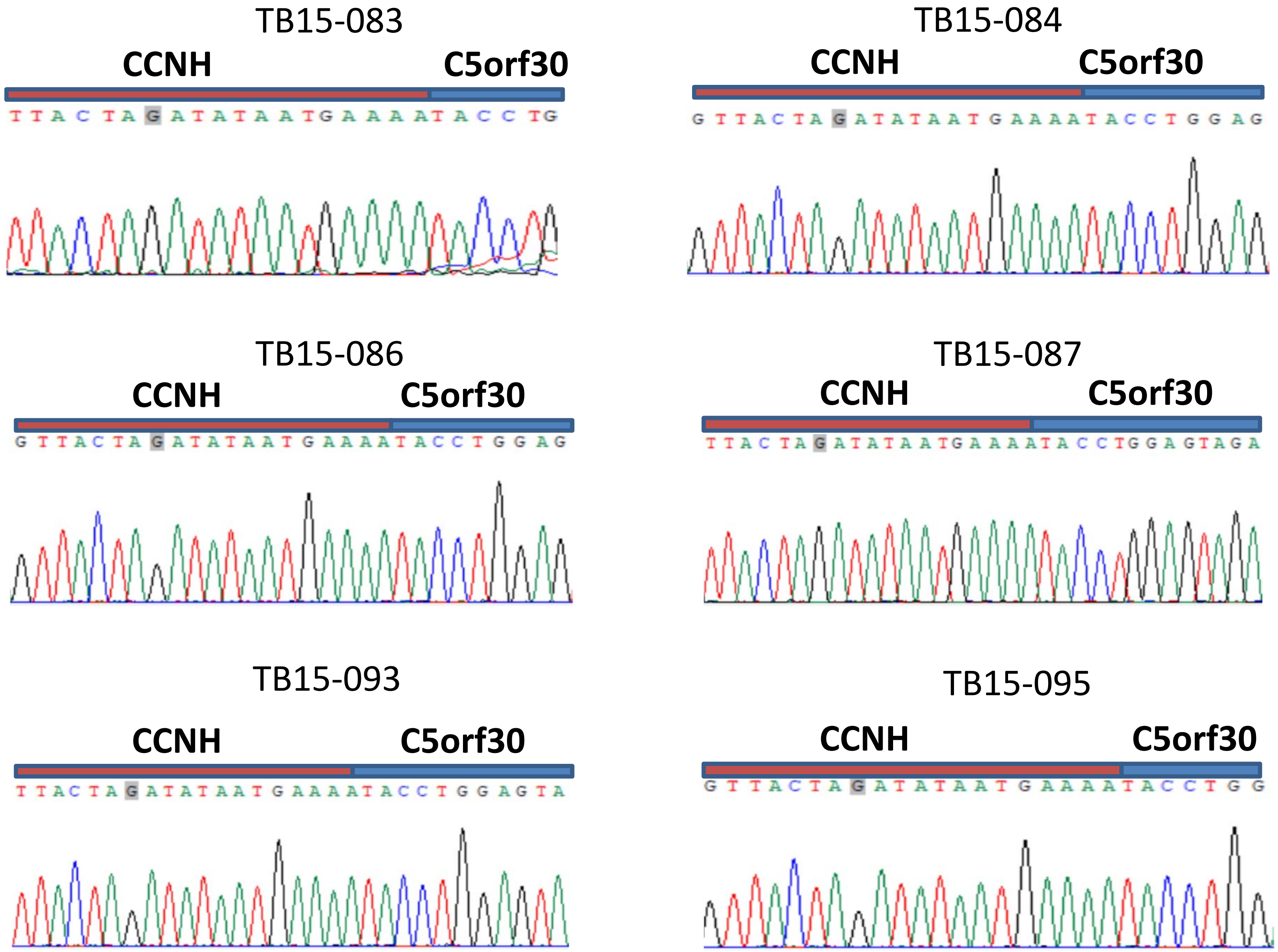
TB15-083
CCNH
C5orf30
TTACTAGATATAATGAAAATACCTG
TB15-084
CCNH
C5orf30
GTTACTAGATATAATGAAAATACCTGGAG
TB15-086
CCNH
C5orf30
GTTACTAGATATAATGAAAATACCTGGAG
TB15-087
CCNH
C5orf30
TTACTAGATATAATGAAAATACCTGGAGTAGA
TB15-093
CCNH
C5orf30
TTACTAGATATAATGAAAATACCTGGAGTA
TB15-095
CCNH
C5orf30
GTTACTAGATATAATGAAAATACCTGG

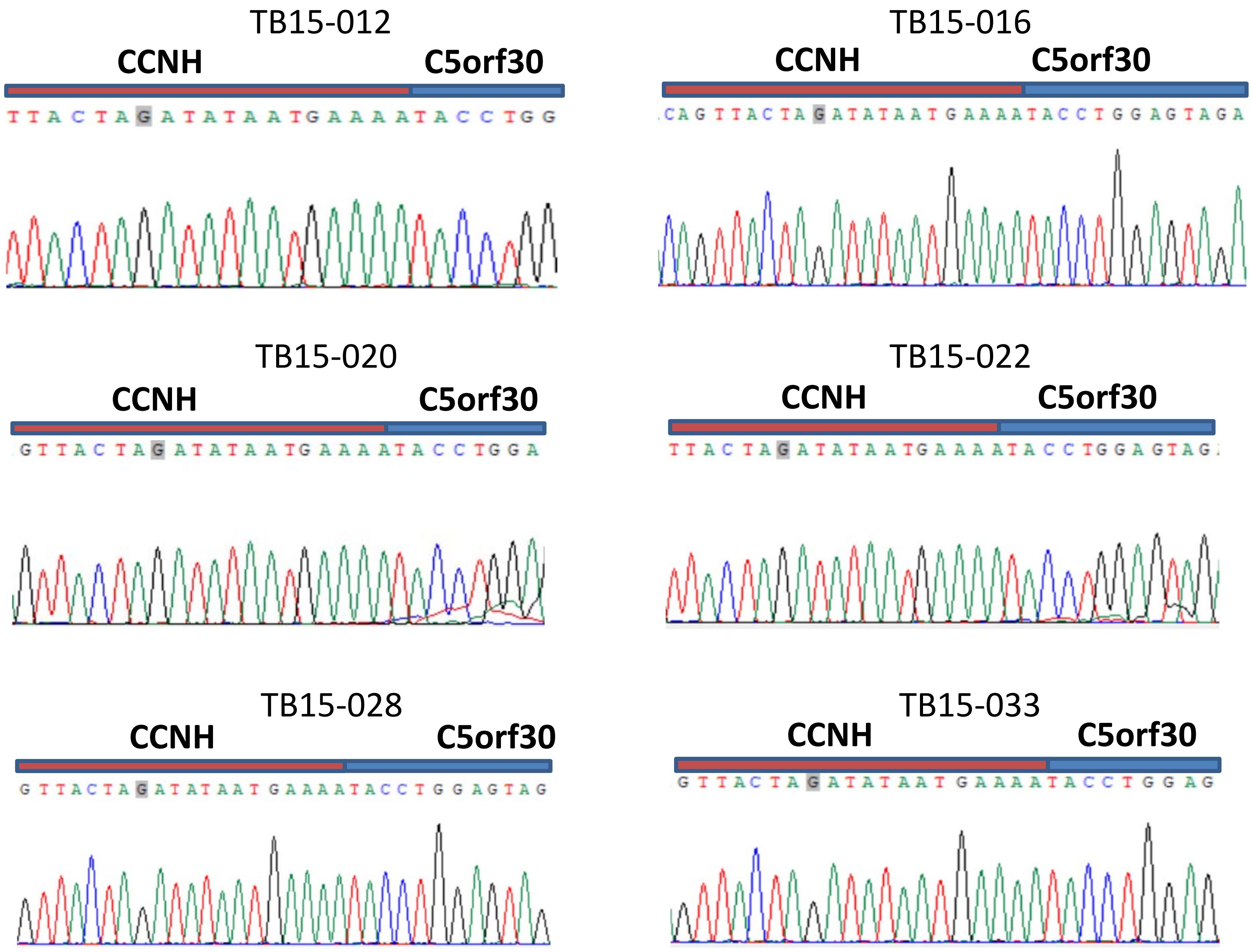
TB15-012
CCNH
C5orf30
TTACTAGATATAATGAAAATACCTGG
TB15-016
CCNH
C5orf30
CAGTTACTAGATATAATGAAAATACCTGGAGTAGA
TB15-020
CCNH
C5orf30
GTTACTAGATATAATGAAAATACCTGGA
TB15-022
CCNH
C5orf30
TTACTAGATATAATGAAAATACCTGGAGTAG
TB15-028
CCNH
C5orf30
GTTACTAGATATAATGAAAATACCTGGAGTAG
TB15-033
CCNH
C5orf30
GTTACTAGATATAATGAAAATACCTGGAG

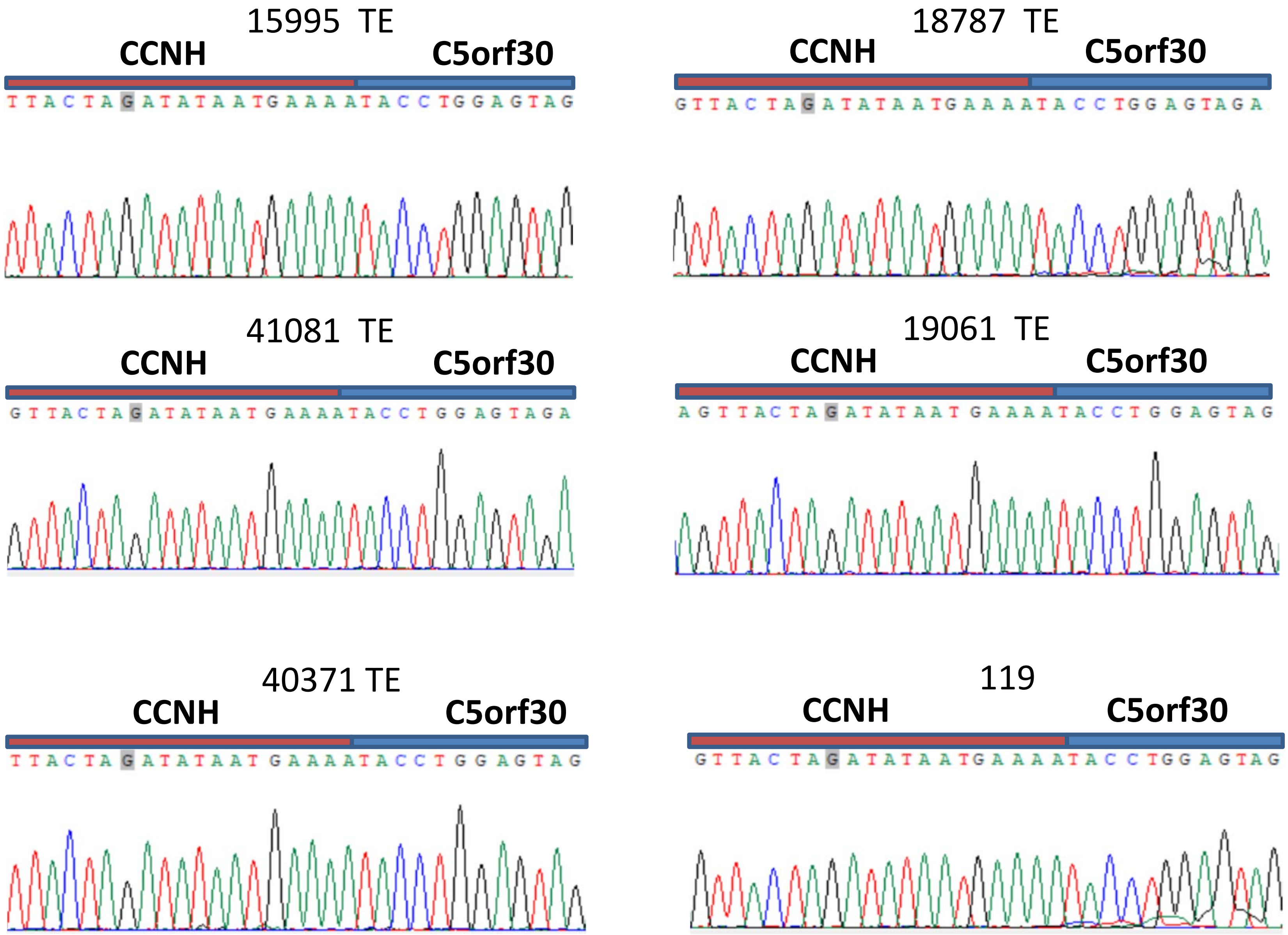
15995 TE
CCNH
C5orf30
TTACTAGATATAATGAAAATACCTGGAGTAG
18787 TE
CCNH
C5orf30
GTTACTAGATATAATGAAAATACCTGGAGTAGA
41081 TE
CCNH
C5orf30
GTTACTAGATATAATGAAAATACCTGGAGTAGA
19061 TE
CCNH
C5orf30
AGTTACTAGATATAATGAAAATACCTGGAGTAG
40371 TE
CCNH
C5orf30
TTACTAGATATAATGAAAATACCTGGAGTAG
119
CCNH
C5orf30
GTTACTAGATATAATGAAAATACCTGGAGTAG

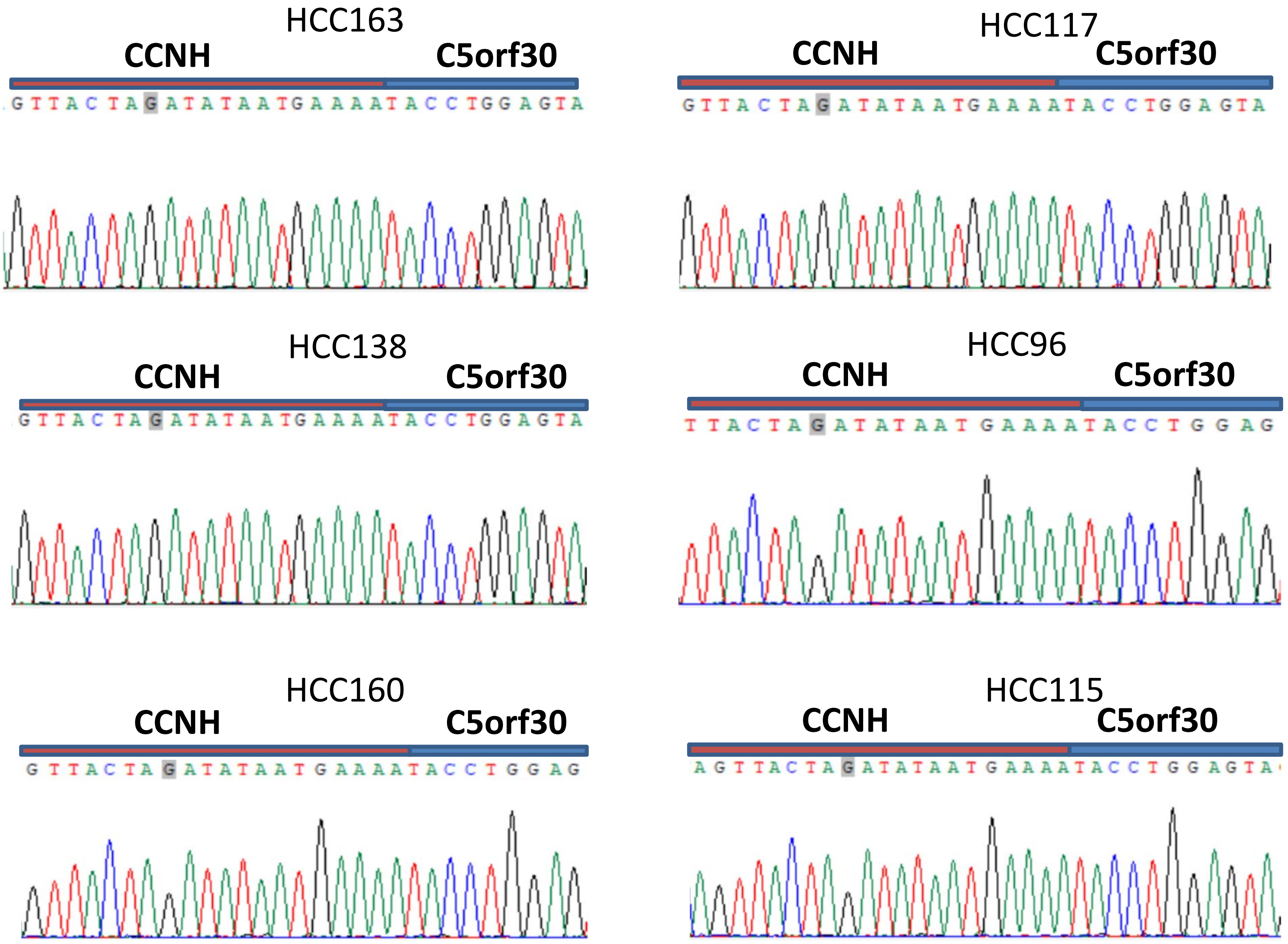
HCC163
CCNH
C5orf30
G T T A C T A G A T A T A A T G A A A A T A C C T G G A G T A
HCC117
CCNH
C5orf30
G T T A C T A G A T A T A A T G A A A A T A C C T G G A G T A
HCC138
CCNH
C5orf30
G T T A C T A G A T A T A A T G A A A A T A C C T G G A G T A
HCC96
CCNH
C5orf30
T T A C T A G A T A T A A T G A A A A T A C C T G G A G
HCC160
CCNH
C5orf30
G T T A C T A G A T A T A A T G A A A A T A C C T G G A G
HCC115
CCNH
C5orf30
A G T T A C T A G A T A T A A T G A A A A T A C C T G G A G T A

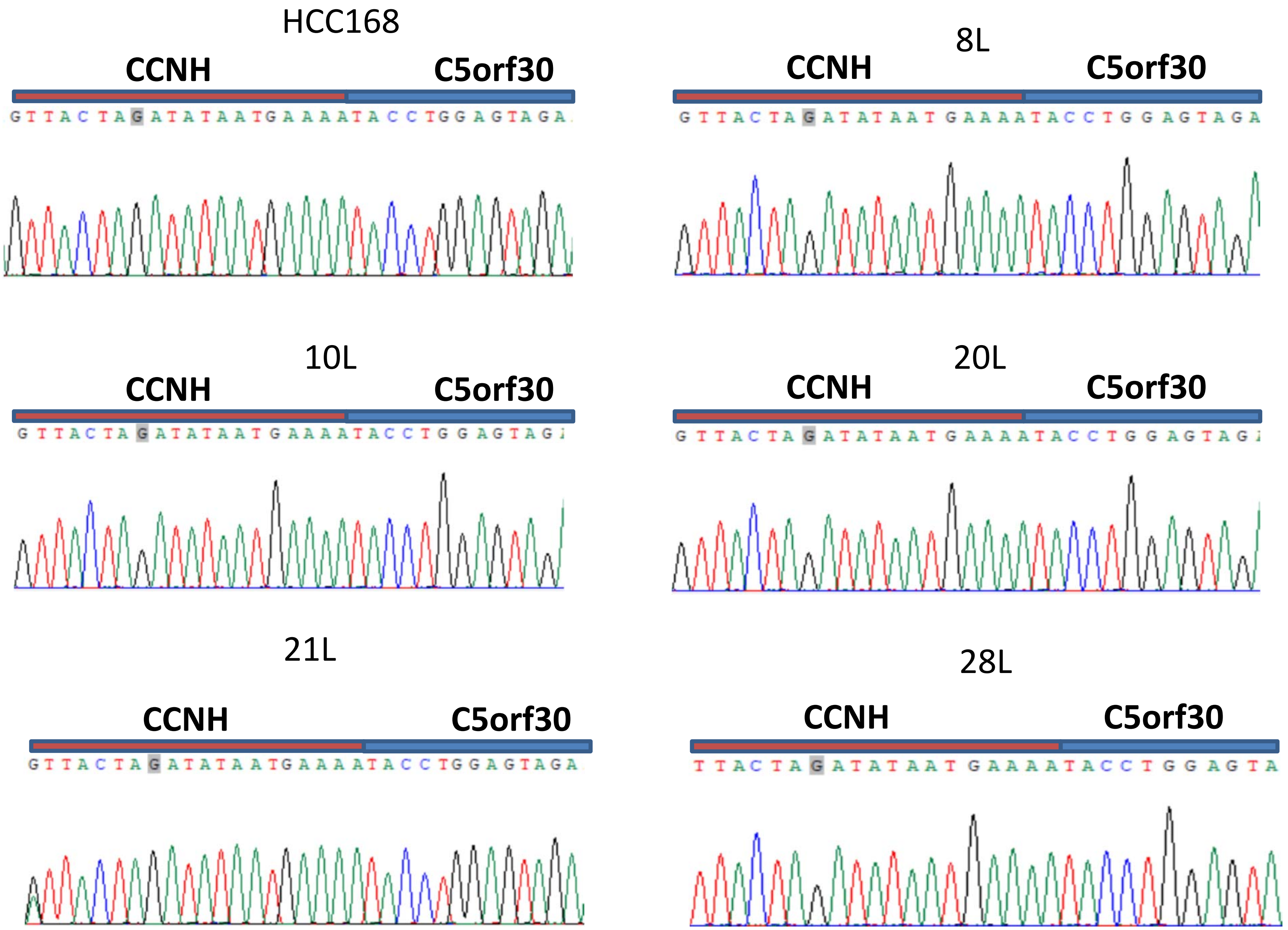
HCC168
CCNH
C5orf30
GTTACTAGATATAATGAAAATACCTGGAGTAGA
8L
CCNH
C5orf30
GTTACTAGATATAATGAAAATACCTGGAGTAGA
10L
CCNH
C5orf30
GTTACTAGATATAATGAAAATACCTGGAGTAG
20L
CCNH
C5orf30
GTTACTAGATATAATGAAAATACCTGGAGTAG
21L
CCNH
C5orf30
GTTACTAGATATAATGAAAATACCTGGAGTAGA
28L
CCNH
C5orf30
TTACTAGATATAATGAAAATACCTGGAGTA

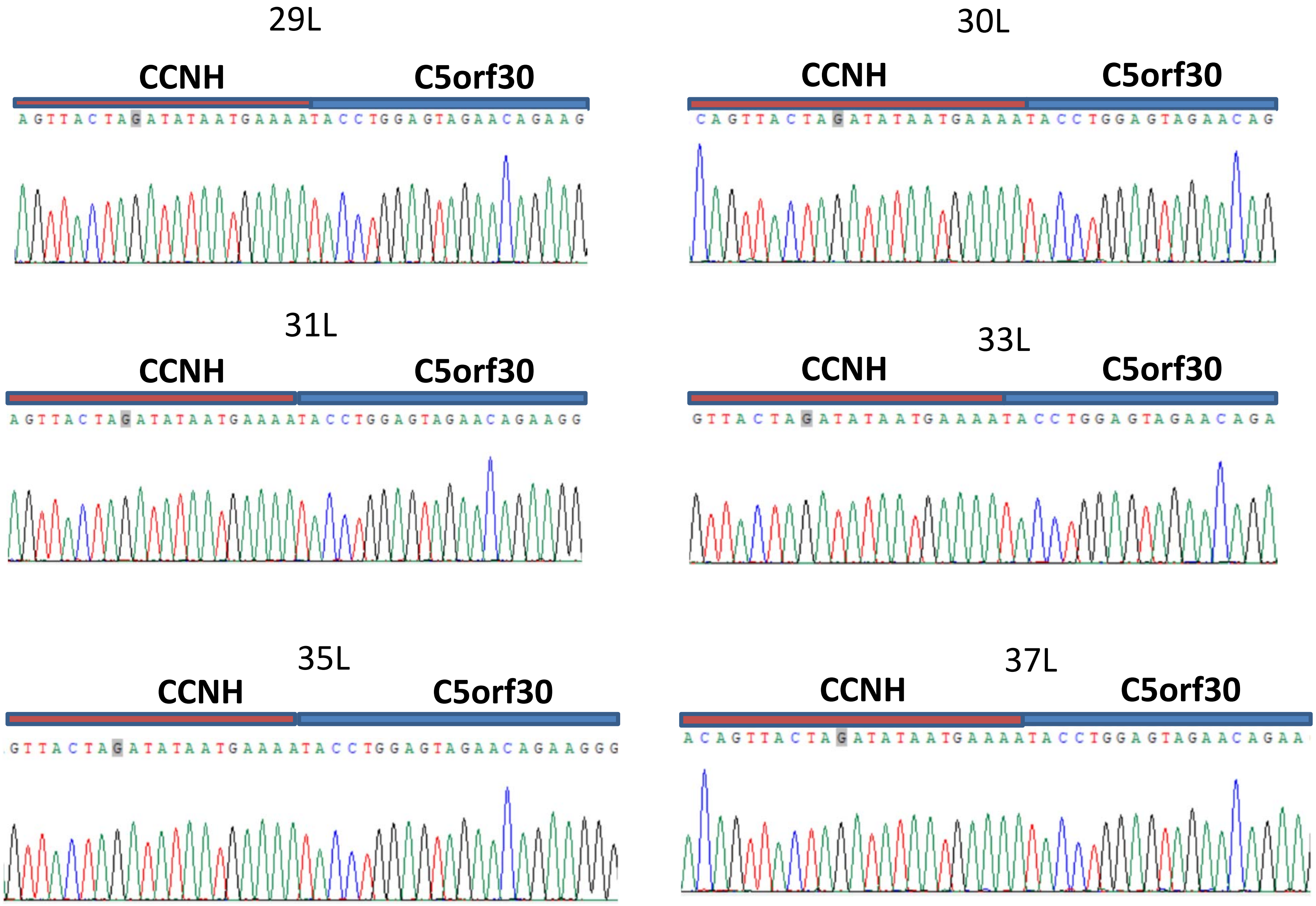
29L
CCNH
C5orf30
AGTTACTAGATATAATGAAAATACCTGGAGTAGAACAGAAG
30L
CCNH
C5orf30
CAGTTACTAGATATAATGAAAATACCTGGAGTAGAACAG
31L
CCNH
C5orf30
AGTTACTAGATATAATGAAAATACCTGGAGTAGAACAGAAGG
33L
CCNH
C5orf30
GTTACTAGATATAATGAAAATACCTGGAGTAGAACAGA
35L
CCNH
C5orf30
GTTACTAGATATAATGAAAATACCTGGAGTAGAACAGAAGGG
37L
CCNH
C5orf30
ACAGTTACTAGATATAATGAAAATACCTGGAGTAGAACAGAA

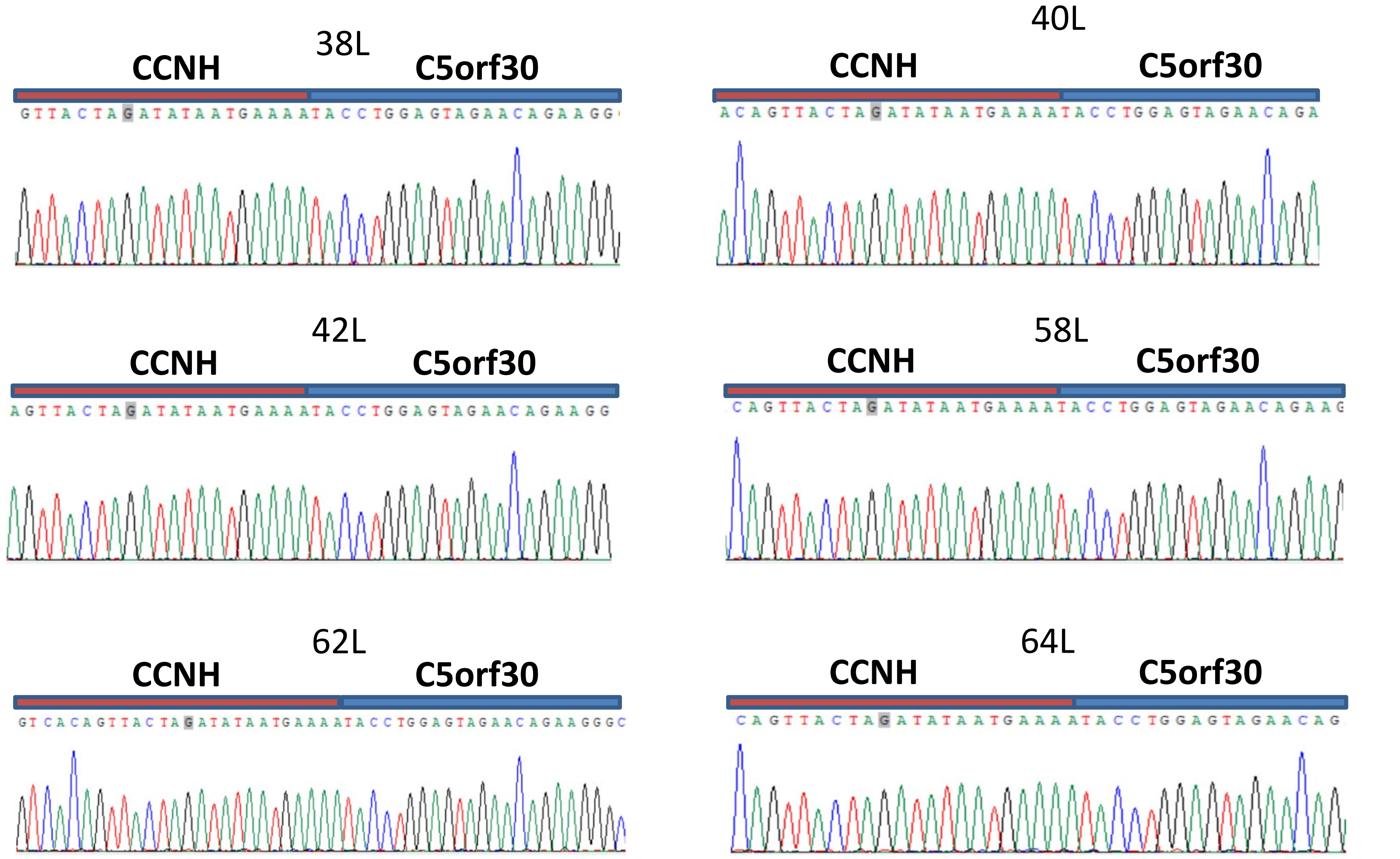

38L
CCNH
C5orf30
GTTACTAGATATAATGAAAATACCTGGAGTAGAACAGAAGG
40L
CCNH
C5orf30
ACAGTTACTAGATATAATGAAAATACCTGGAGTAGAACAGA
42L
CCNH
C5orf30
AGTTACTAGATATAATGAAAATACCTGGAGTAGAACAGAAGG
58L
CCNH
C5orf30
CAGTTACTAGATATAATGAAAATACCTGGAGTAGAACAGAAG
62L
CCNH
C5orf30
GTCACAGTTACTAGATATAATGAAAATACCTGGAGTAGAACAGAAGGGC
64L
CCNH
C5orf30
CAGTTACTAGATATAATGAAAATACCTGGAGTAGAACAG

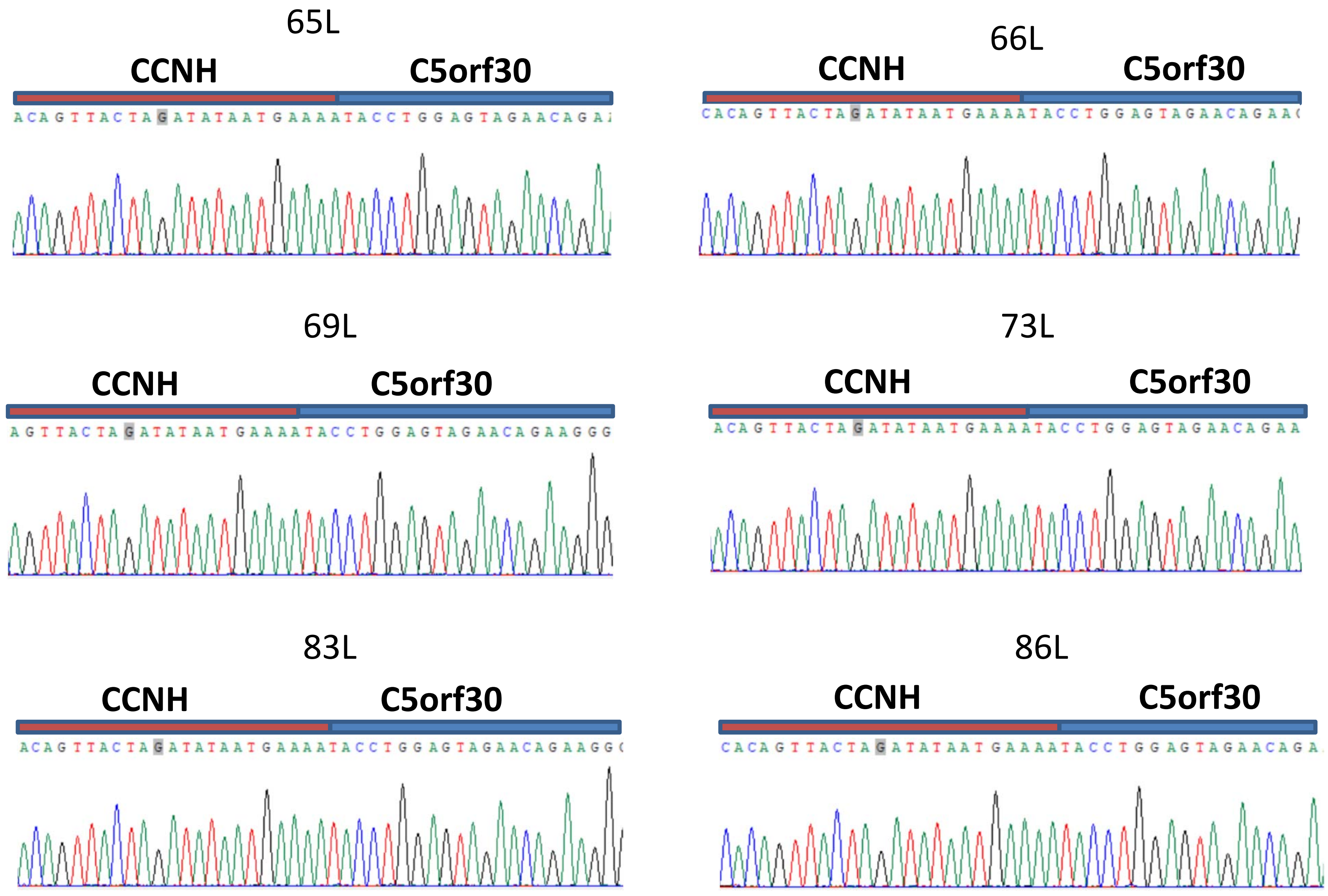
65L
CCNH
C5orf30
ACAGTTACTAGATATAATGAAAATACCTGGAGTAGAACAGA
66L
CCNH
C5orf30
CACAGTTACTAGATATAATGAAAATACCTGGAGTAGAACAGAA
69L
CCNH
C5orf30
AGTTACTAGATATAATGAAAATACCTGGAGTAGAACAGAAGGG
73L
CCNH
C5orf30
ACAGTTACTAGATATAATGAAAATACCTGGAGTAGAACAGAA
83L
CCNH
C5orf30
ACAGTTACTAGATATAATGAAAATACCTGGAGTAGAACAGAAGG
86L
CCNH
C5orf30
CACAGTTACTAGATATAATGAAAATACCTGGAGTAGAACAGA

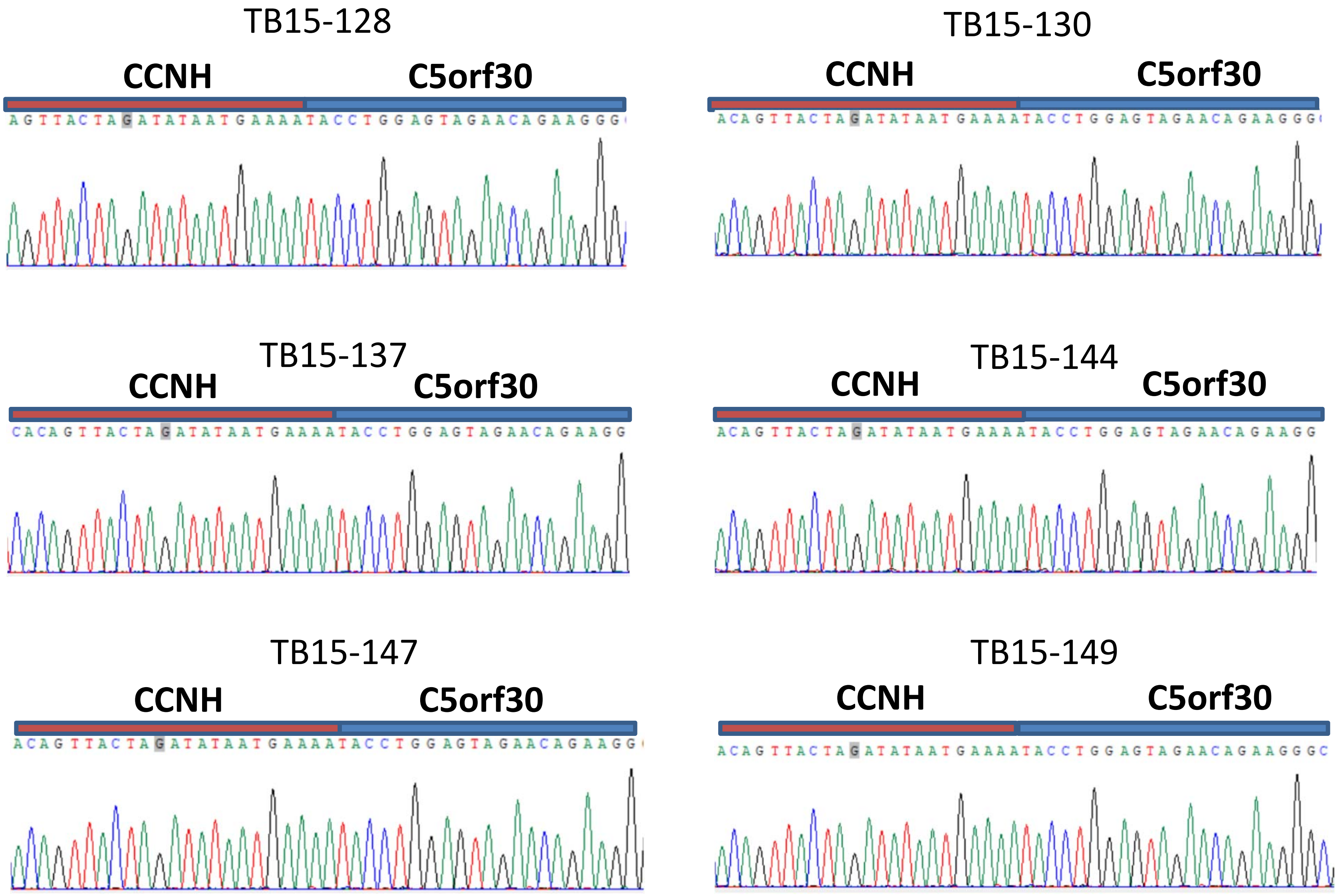
TB15-128
CCNH
C5orf30
AGTTACTAGATATAATGAAAATACCTGGAGTAGAACAGAAGGG
TB15-130
CCNH
C5orf30
ACAGTTACTAGATATAATGAAAATACCTGGAGTAGAACAGAAGGG
TB15-137
CCNH
C5orf30
CACAGTTACTAGATATAATGAAAATACCTGGAGTAGAACAGAAGG
TB15-144
CCNH
C5orf30
ACAGTTACTAGATATAATGAAAATACCTGGAGTAGAACAGAAGG
TB15-147
CCNH
C5orf30
ACAGTTACTAGATATAATGAAAATACCTGGAGTAGAACAGAAGG
TB15-149
CCNH
C5orf30
ACAGTTACTAGATATAATGAAAATACCTGGAGTAGAACAGAAGGGC

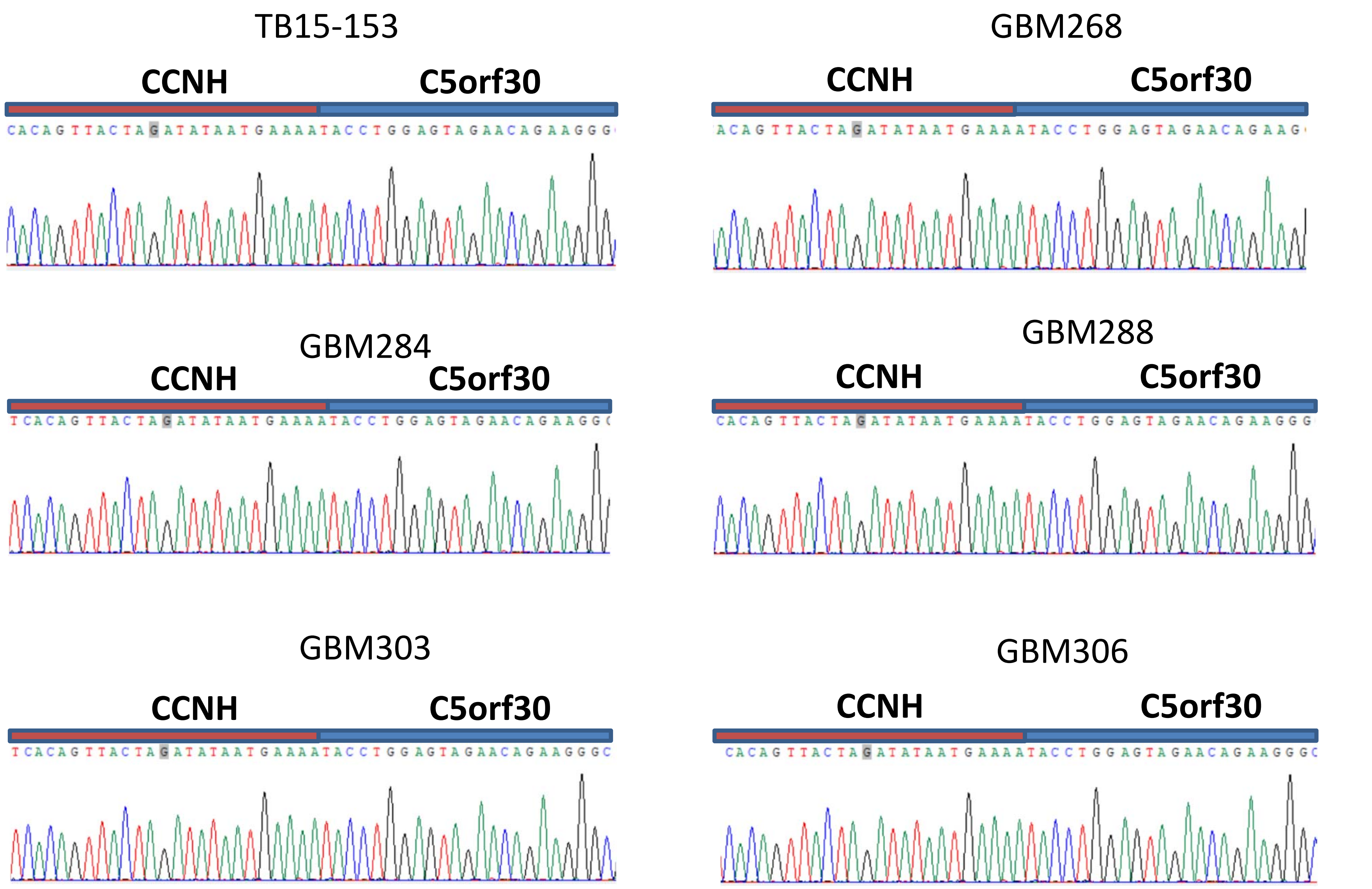
TB15-153
CCNH
C5orf30
GBM268
CCNH
C5orf30
GBM284
CCNH
C5orf30
GBM288
CCNH
C5orf30
GBM303
CCNH
C5orf30
GBM306
CCNH
C5orf30

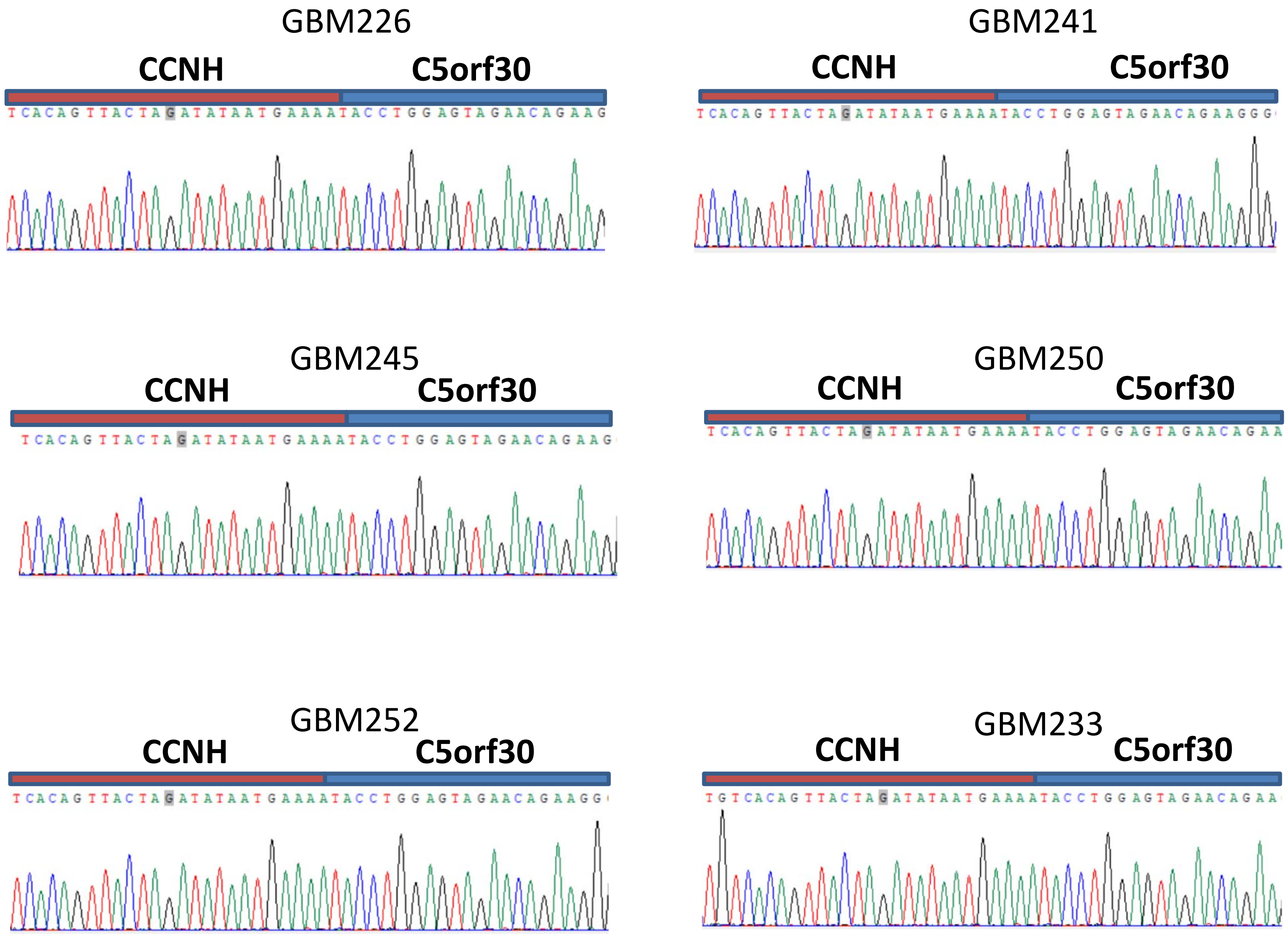
GBM226
CCNH
C5orf30
TCACAGTTACTAGATATAATGAAAATACCTGGAGTAGAACAGAAG
GBM241
CCNH
C5orf30
TCACAGTTACTAGATATAATGAAAATACCTGGAGTAGAACAGAAGGG
GBM245
CCNH
C5orf30
TCACAGTTACTAGATATAATGAAAATACCTGGAGTAGAACAGAAG
GBM250
CCNH
C5orf30
TCACAGTTACTAGATATAATGAAAATACCTGGAGTAGAACAGAA
GBM252
CCNH
C5orf30
TCACAGTTACTAGATATAATGAAAATACCTGGAGTAGAACAGAAGG
GBM233
CCNH
C5orf30
TGTCACAGTTACTAGATATAATGAAAATACCTGGAGTAGAACAGAA

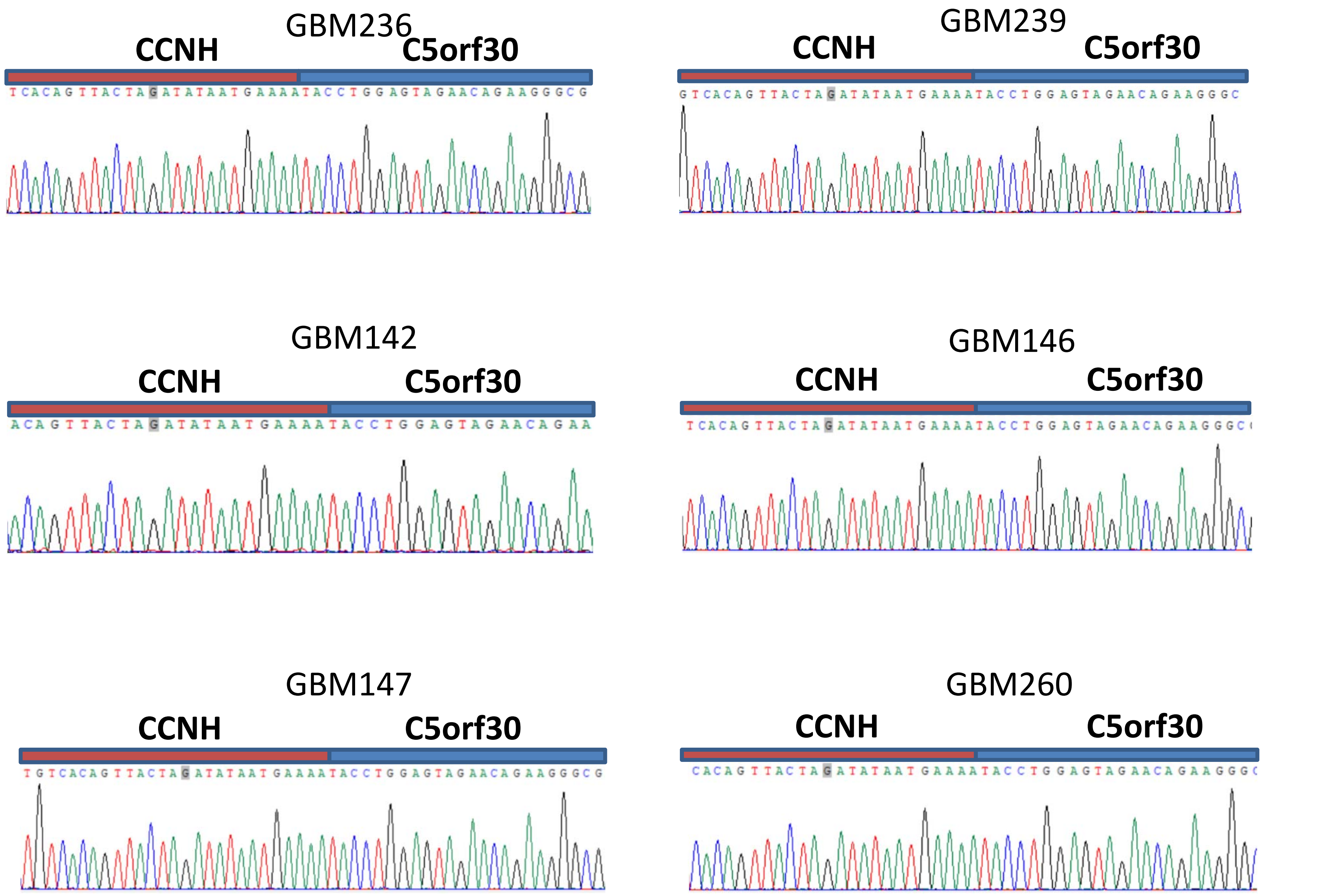
GBM236
CCNH
C5orf30
GBM239
CCNH
C5orf30
GBM142
CCNH
C5orf30
GBM146
CCNH
C5orf30
GBM147
CCNH
C5orf30
GBM260
CCNH
C5orf30

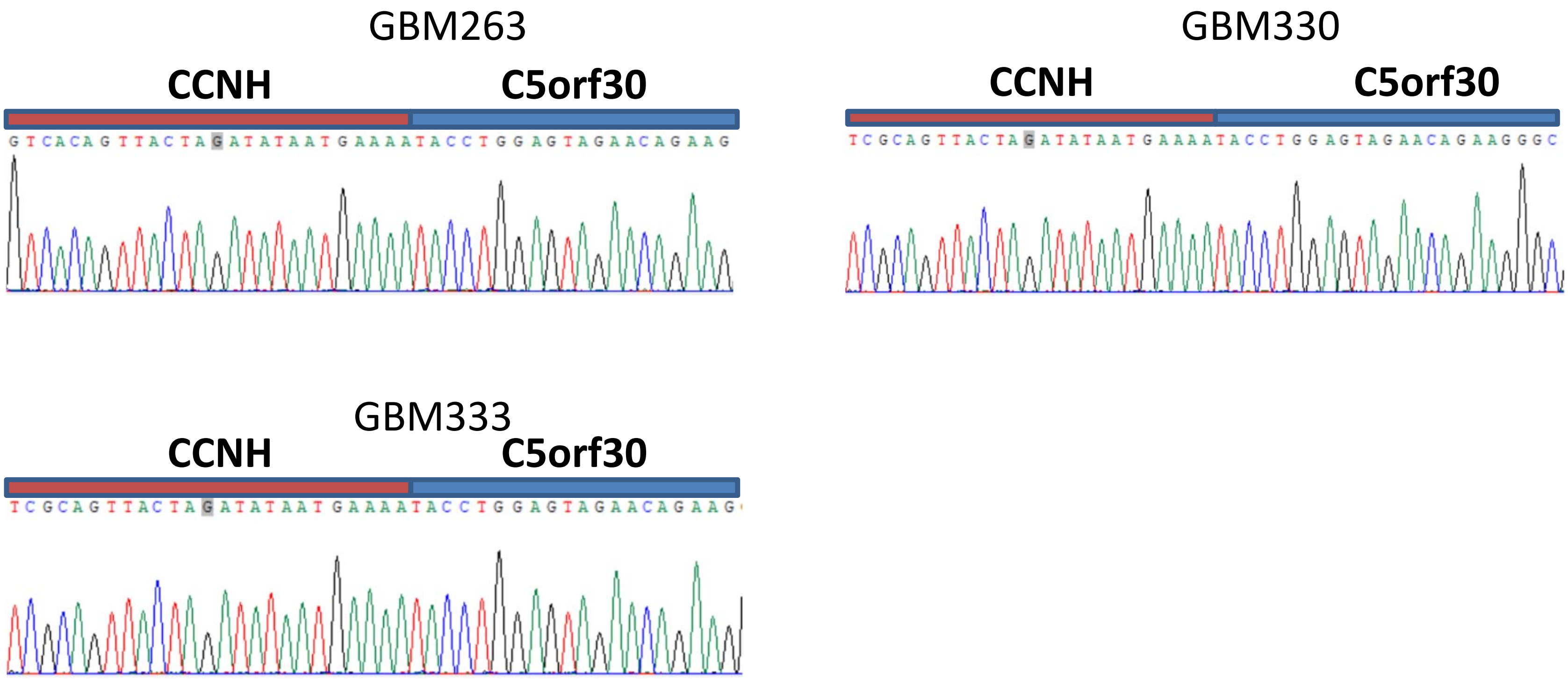
GBM263
CCNH
C5orf30
GTCACAGTTACTAGATATAATGAAAATACCTGGAGTAGAACAGAAG
GBM330
CCNH
C5orf30
TCGCAGTTACTAGATATAATGAAAATACCTGGAGTAGAACAGAAGGGC
GBM333
CCNH
C5orf30
TCGCAGTTACTAGATATAATGAAAATACCTGGAGTAGAACAGAAG

Supplemental figure 2A.

TRMT11-GRIK2 fusion
Cell lines

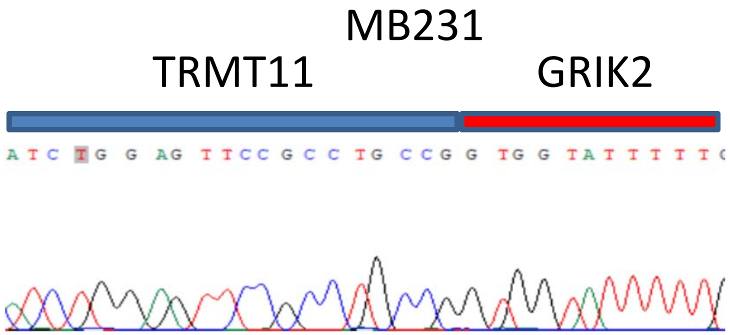

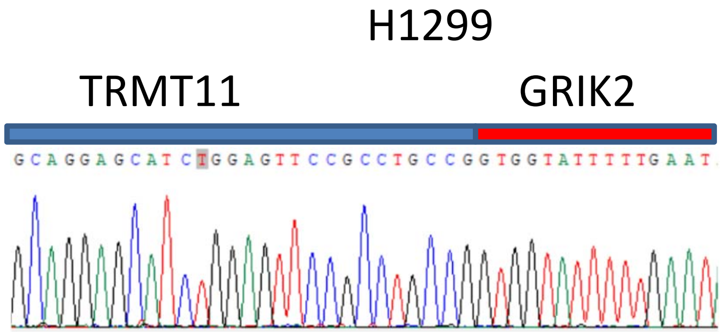

Supplemental figure 2B.

TRMT11-GRIK2 fusion
Clinical specimens

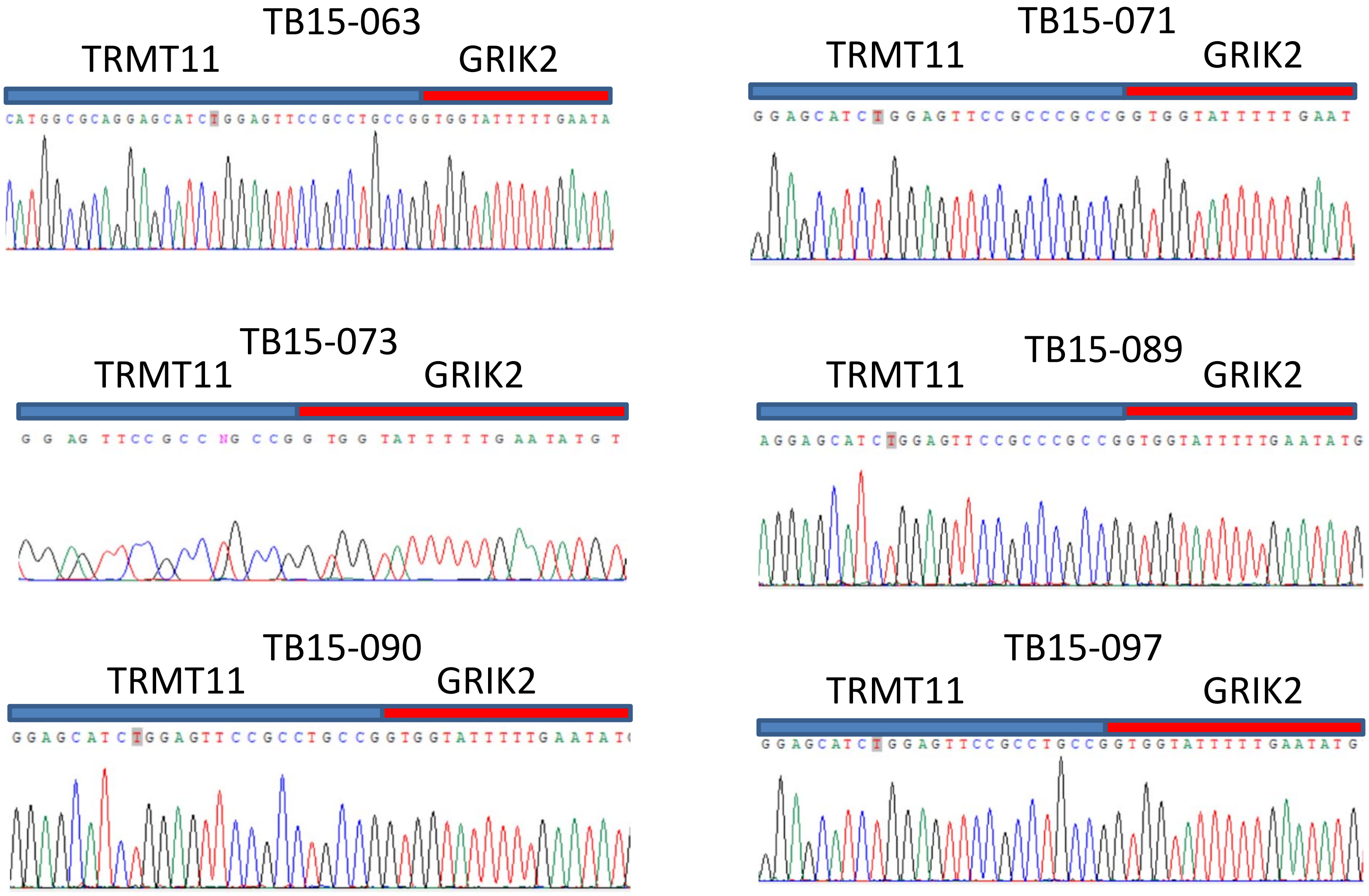

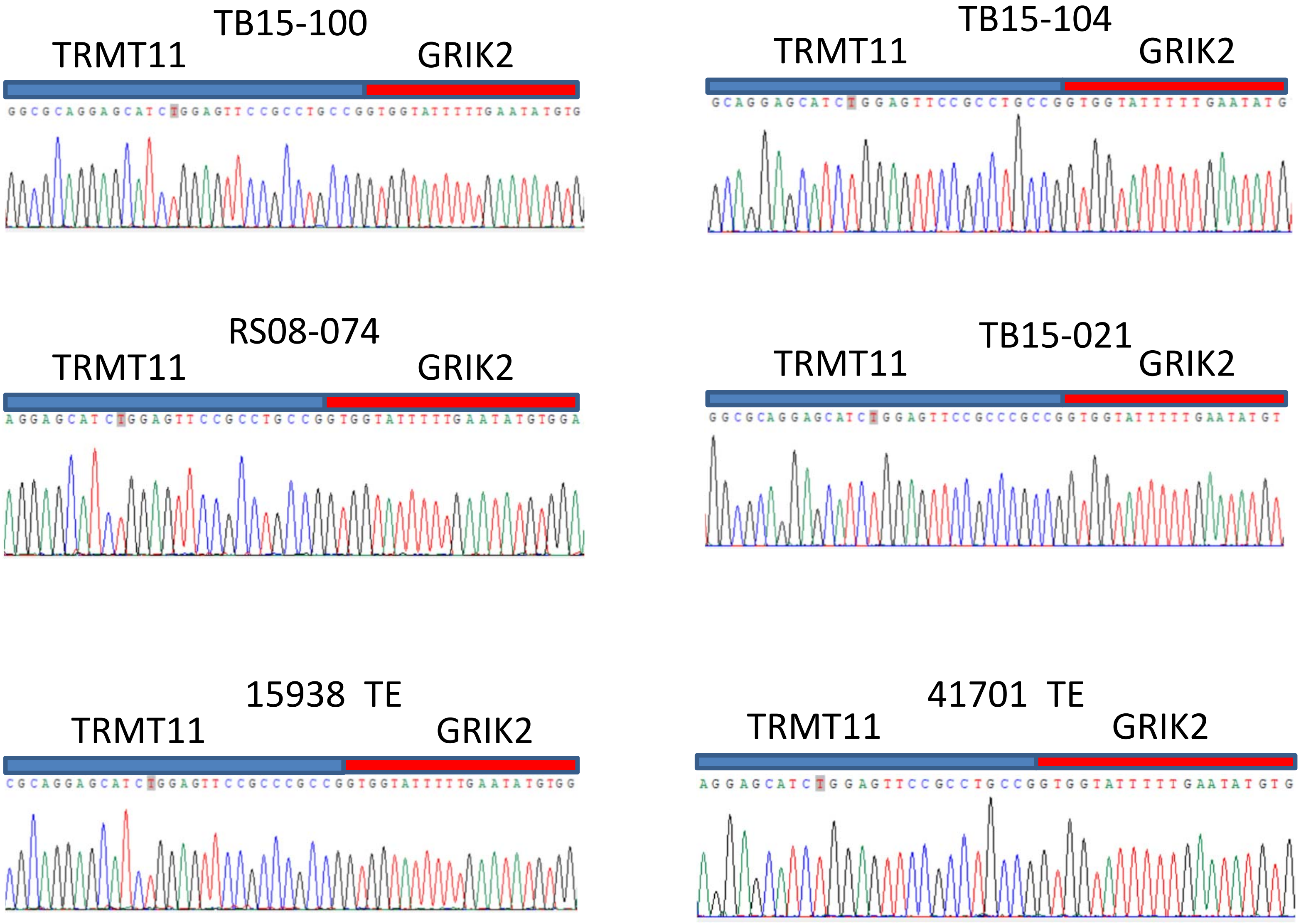
TB15-100
TRMT11
GRIK2
TB15-104
TRMT11
GRIK2
RS08-074
TRMT11
GRIK2
TB15-021
TRMT11
GRIK2
15938 TE
TRMT11
GRIK2
41701 TE
TRMT11
GRIK2

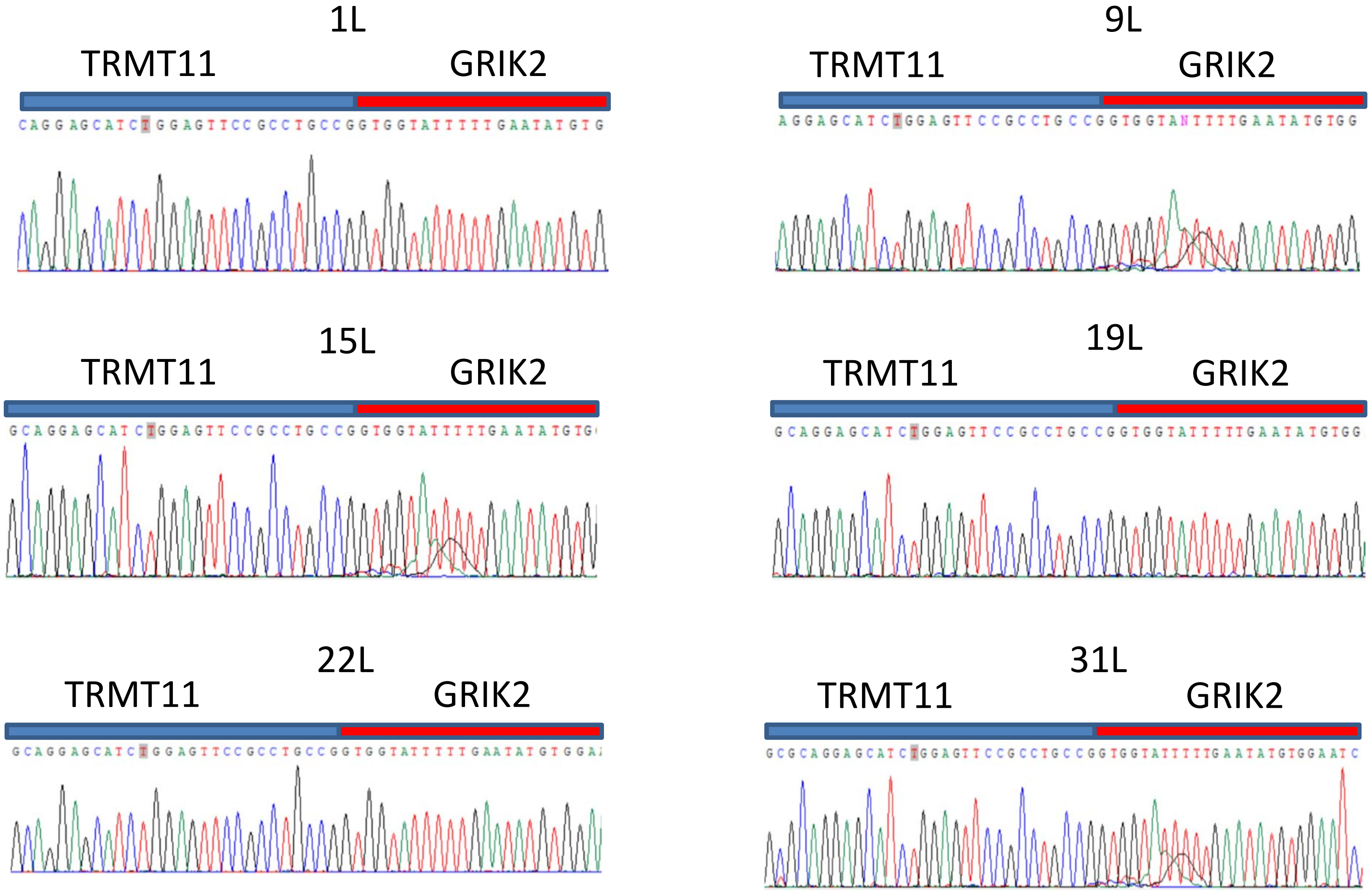
1L
TRMT11
GRIK2
9L
TRMT11
GRIK2
15L
TRMT11
GRIK2
19L
TRMT11
GRIK2
22L
TRMT11
GRIK2
31L
TRMT11
GRIK2

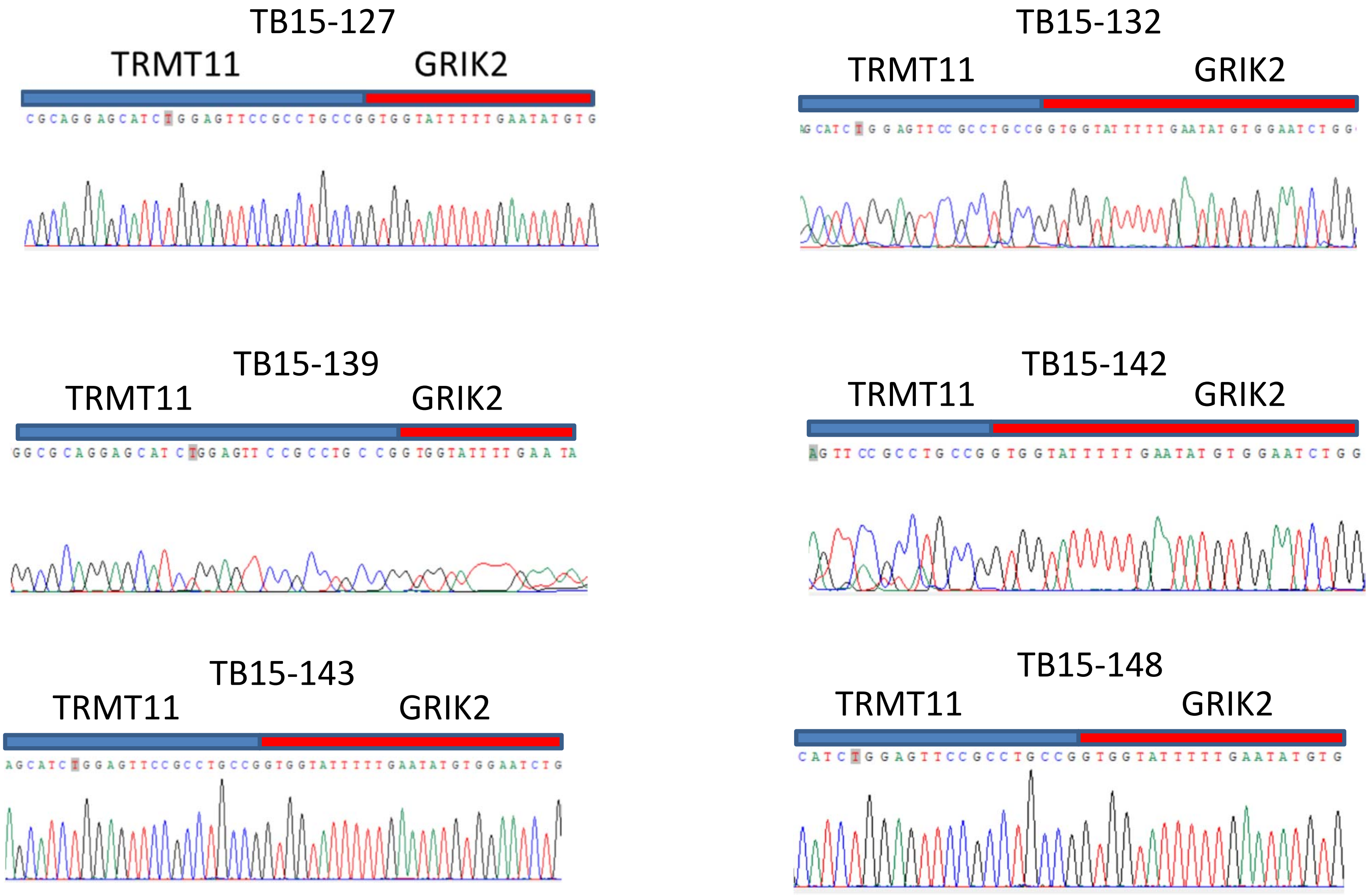
TB15-127
TRMT11
GRIK2
TB15-132
TRMT11
GRIK2
TB15-139
TRMT11
GRIK2
TB15-142
TRMT11
GRIK2
TB15-143
TRMT11
GRIK2
TB15-148
TRMT11
GRIK2

TB15-151

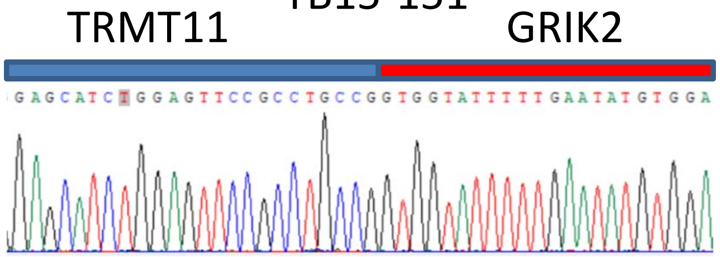

TB15-153

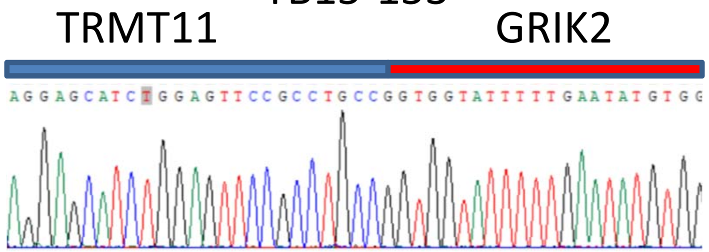

TB15-157

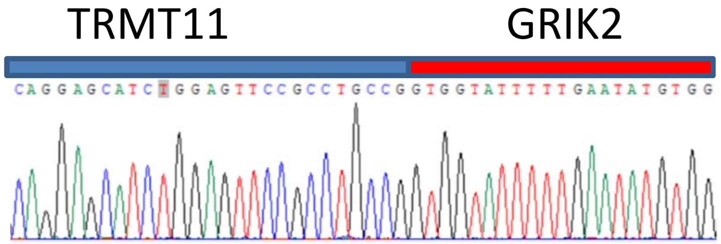

TB15-180

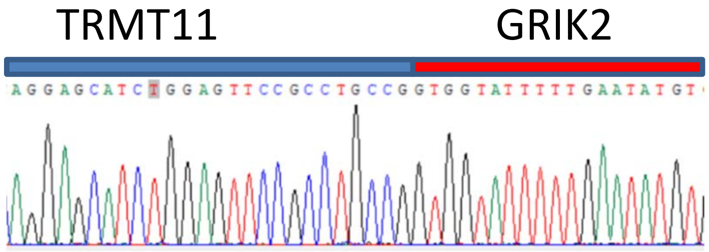

TB15-181

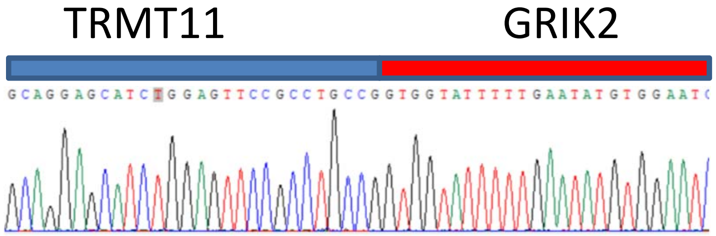

TB15-186

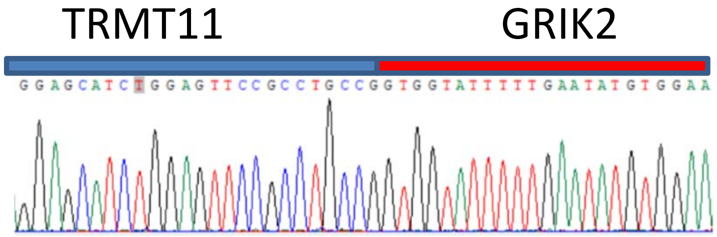

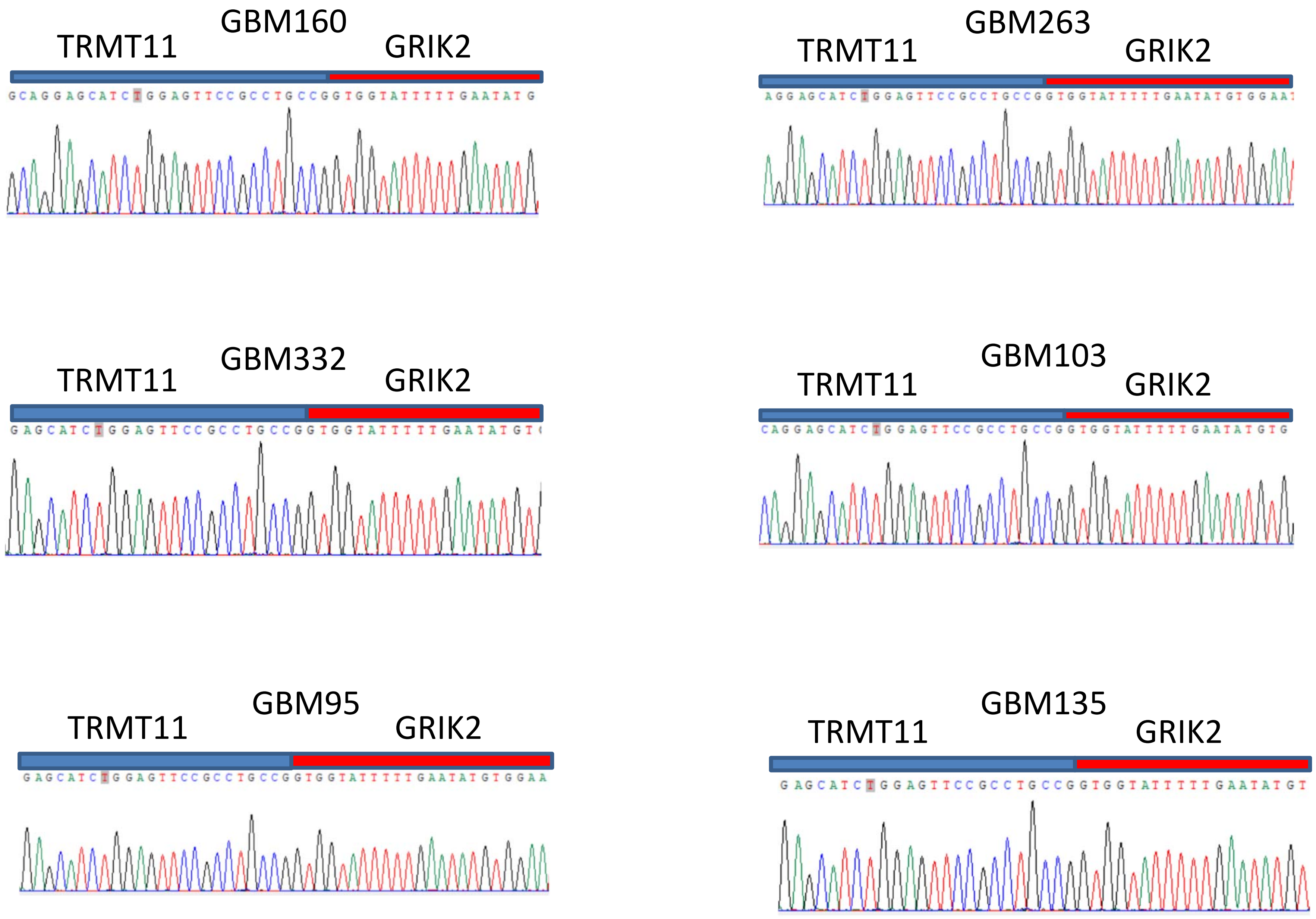
GBM160
TRMT11
GRIK2
GBM263
TRMT11
GRIK2
GBM332
TRMT11
GRIK2
GBM103
TRMT11
GRIK2
GBM95
TRMT11
GRIK2
GBM135
TRMT11
GRIK2

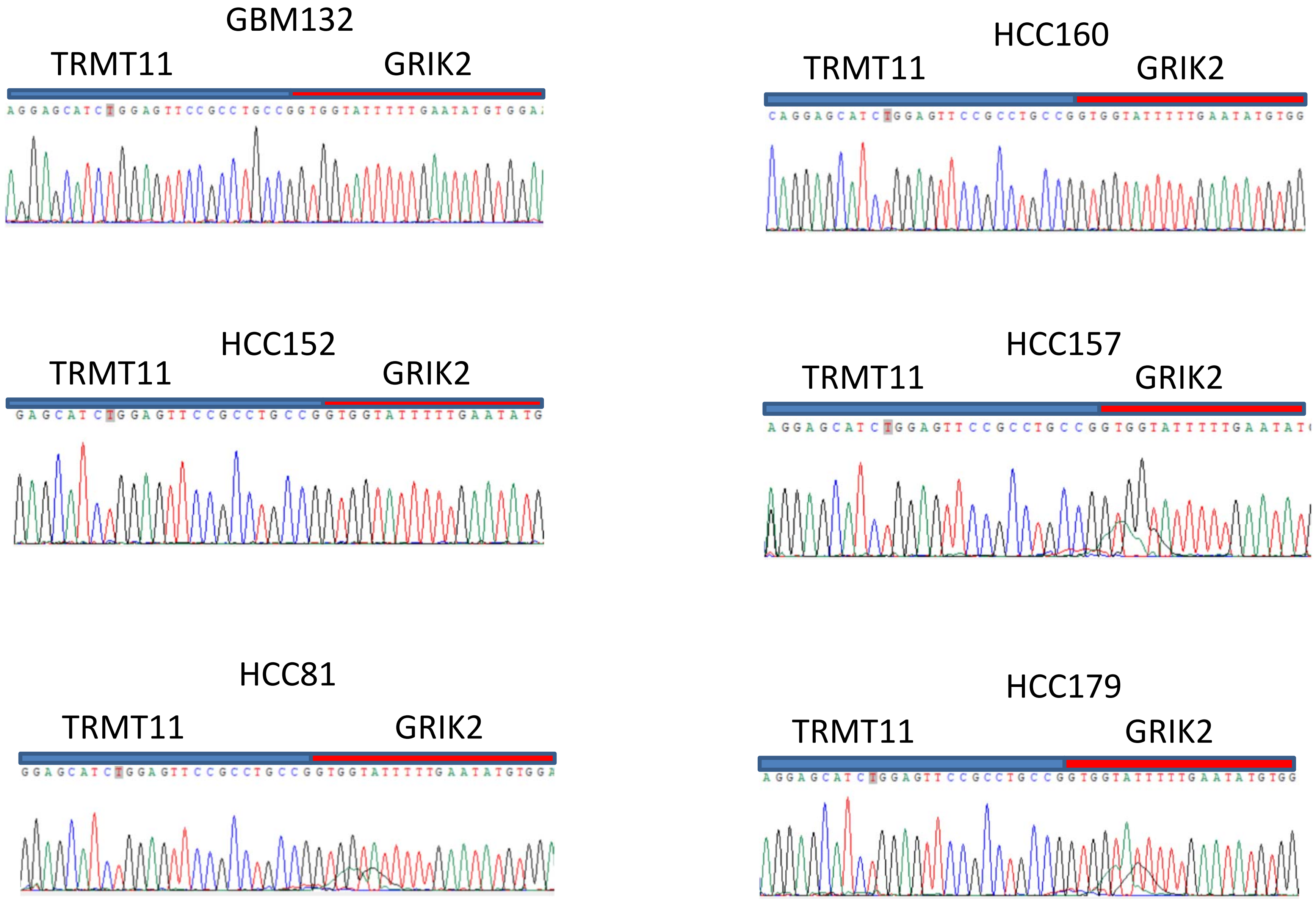

GBM132
TRMT11
GRIK2
HCC160
TRMT11
GRIK2
HCC152
TRMT11
GRIK2
HCC157
TRMT11
GRIK2
HCC81
TRMT11
GRIK2
HCC179
TRMT11
GRIK2

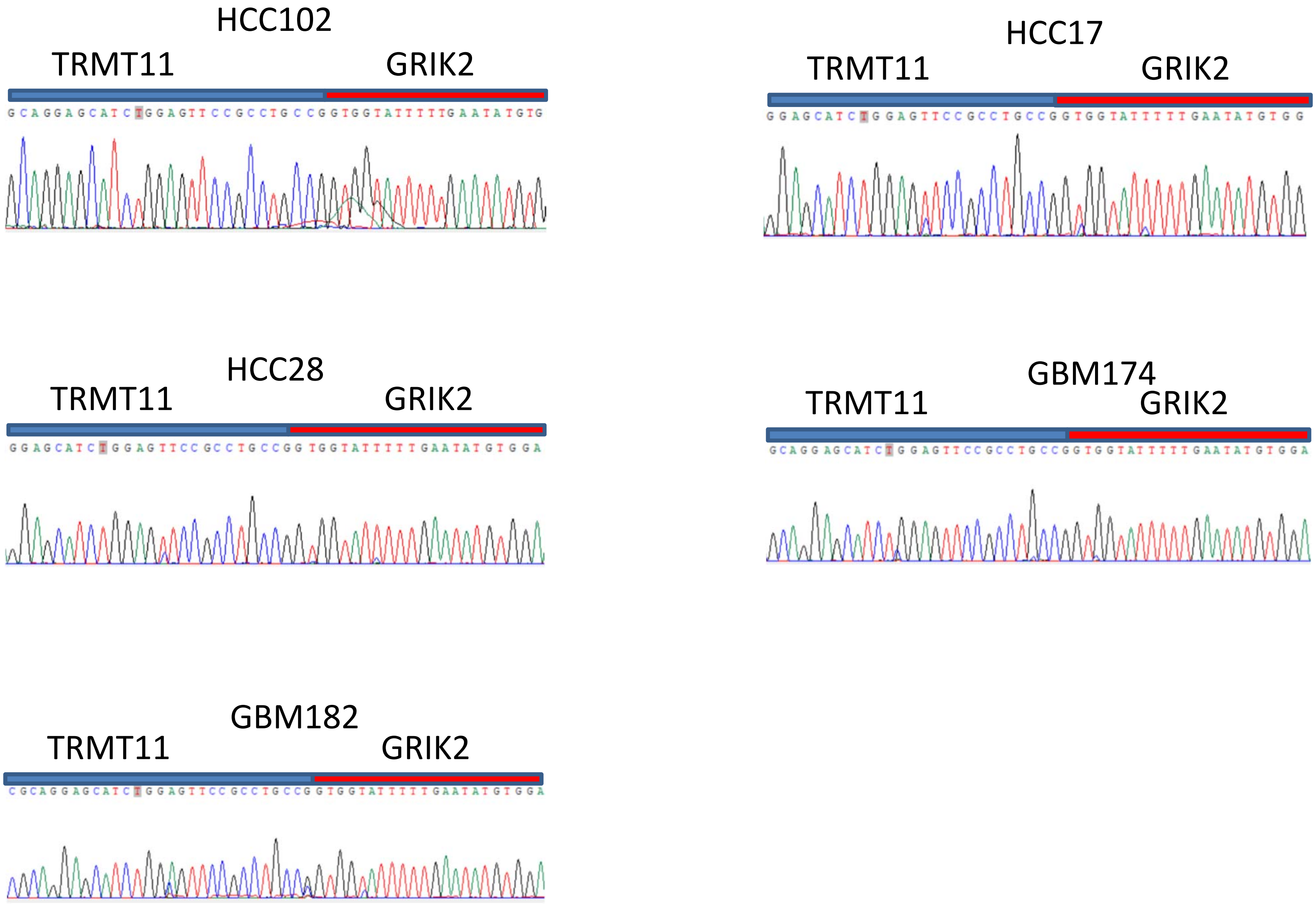
HCC102
TRMT11
GRIK2
HCC17
TRMT11
GRIK2
HCC28
TRMT11
GRIK2
GBM174
TRMT11
GRIK2
GBM182
TRMT11
GRIK2

Supplemental figure 3A. Cell lines

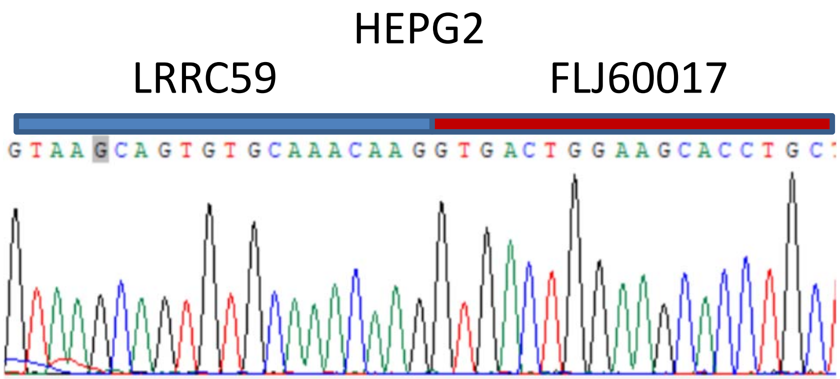

Supplemental figure 3B.

LRRC59-FLJ60017 fusion
Clinical specimens

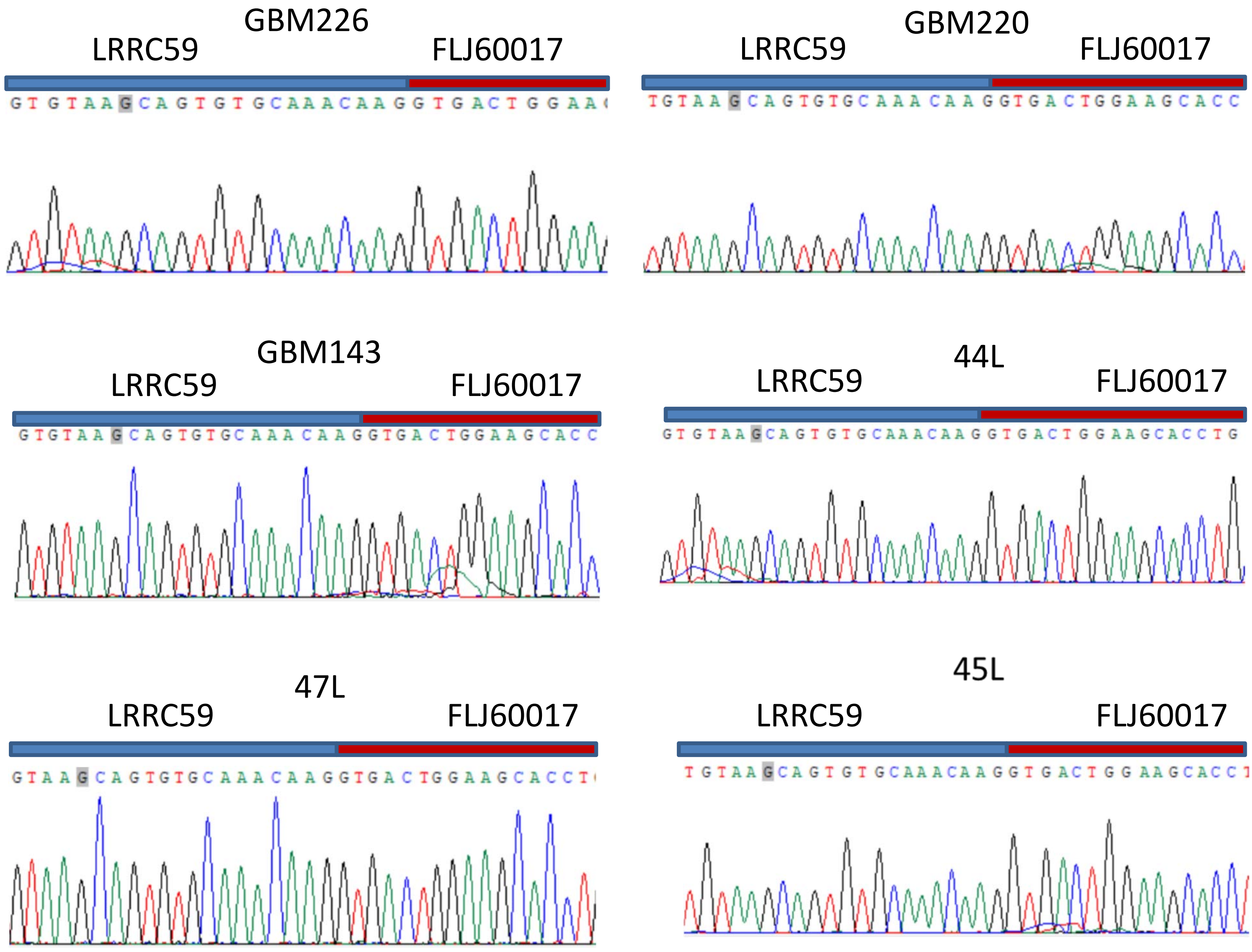

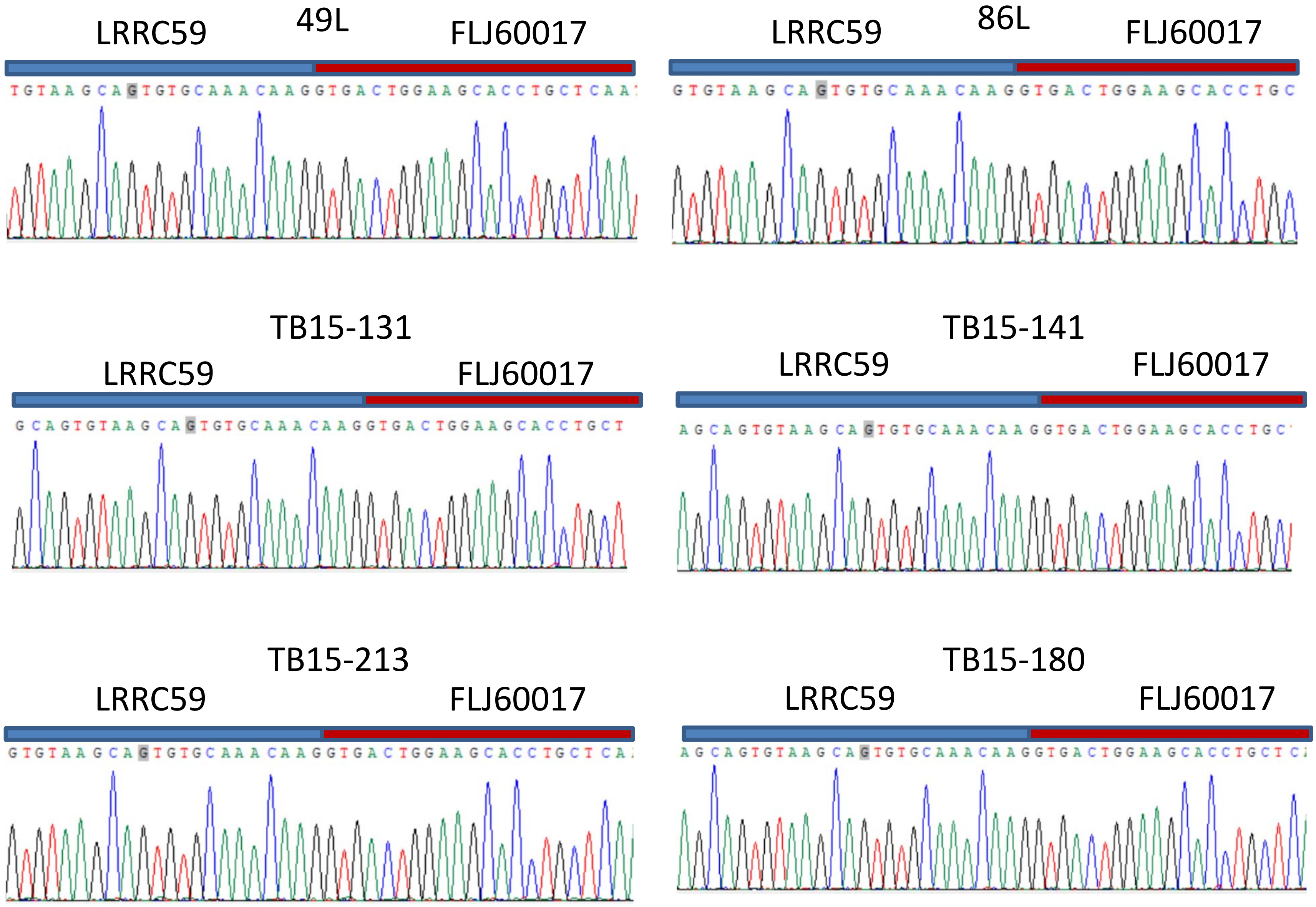
49L
LRRC59
FLJ60017
86L
LRRC59
FLJ60017
TB15-131
LRRC59
FLJ60017
TB15-141
LRRC59
FLJ60017
TB15-213
LRRC59
FLJ60017
TB15-180
LRRC59
FLJ60017

Supplemental figure 4.

TMEM135-CCDC67 fusion
Clinical specimens

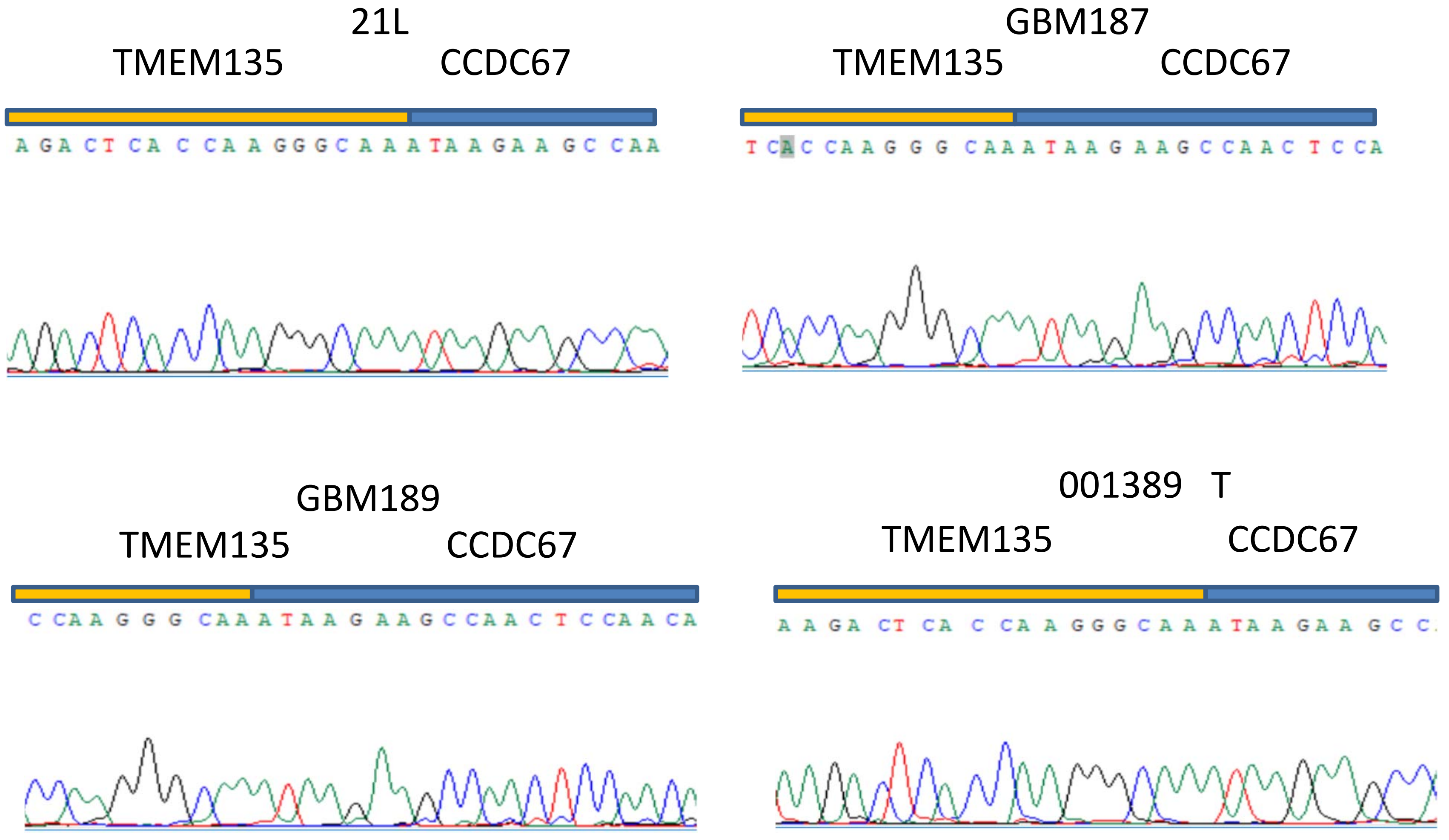

Supplemental figure 5.

mTOR-TP53BP1 fusion
Clinical specimens

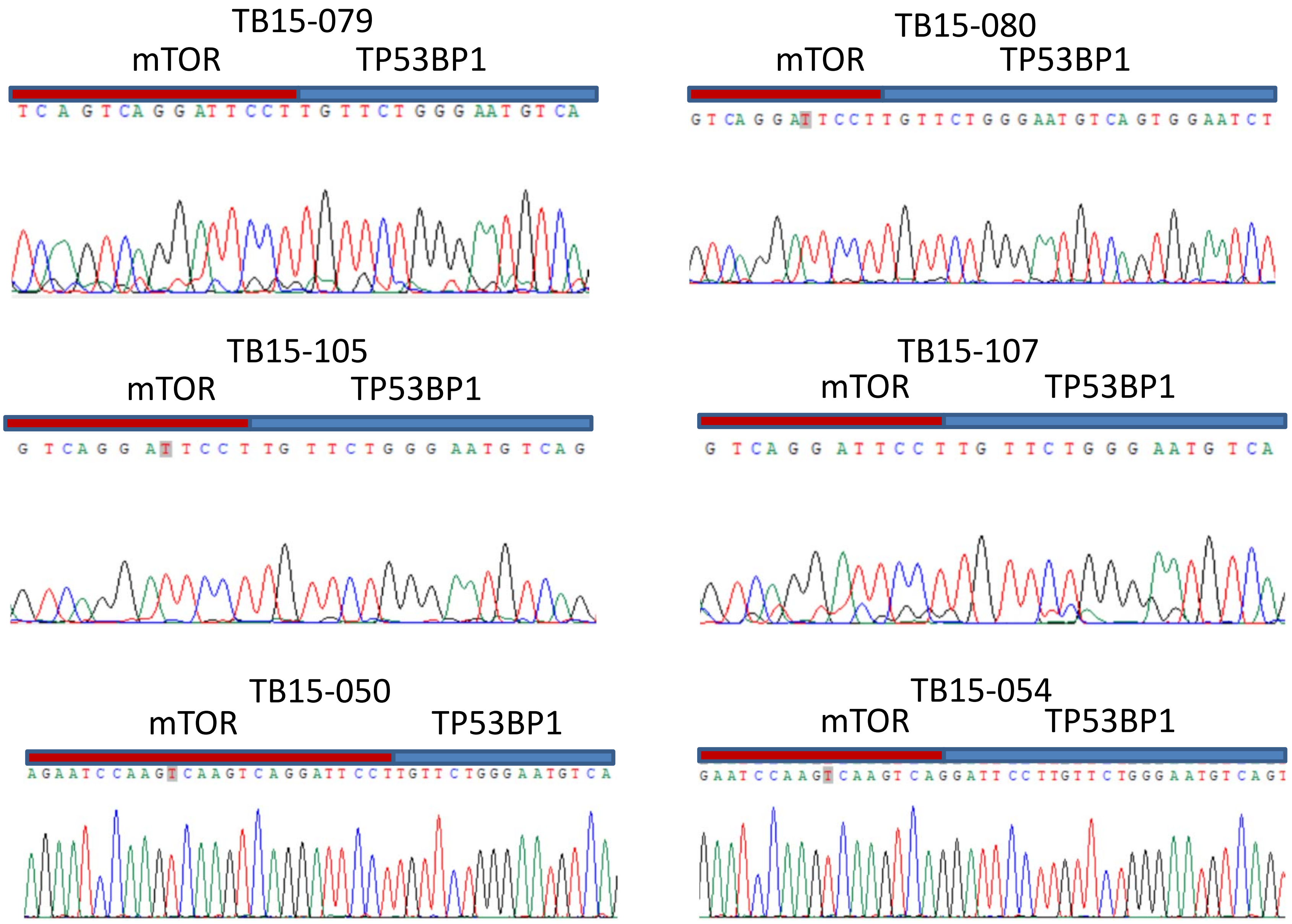

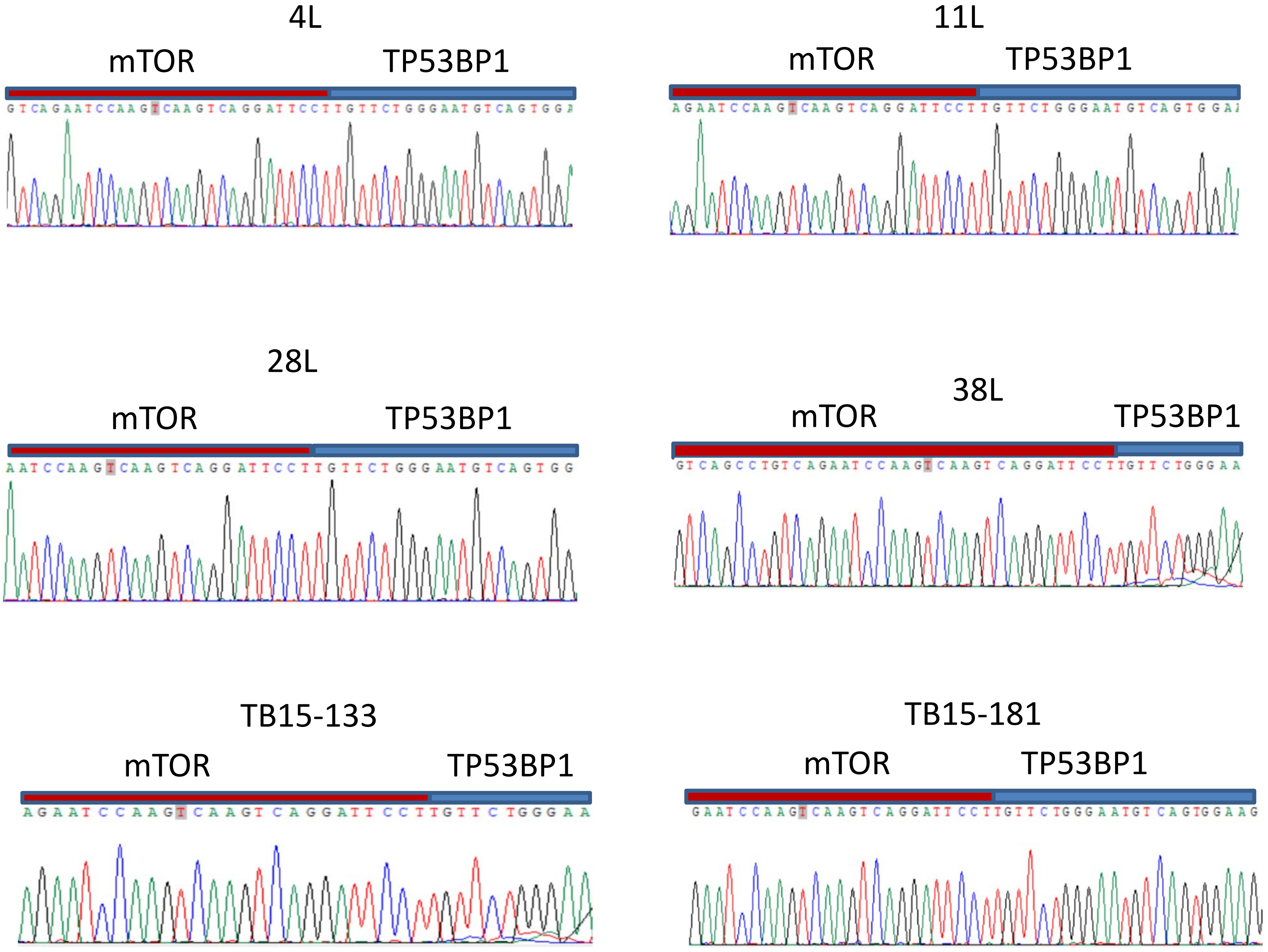
4L
mTOR
TP53BP1
11L
mTOR
TP53BP1
28L
mTOR
TP53BP1
38L
mTOR
TP53BP1
TB15-133
mTOR
TP53BP1
TB15-181
mTOR
TP53BP1

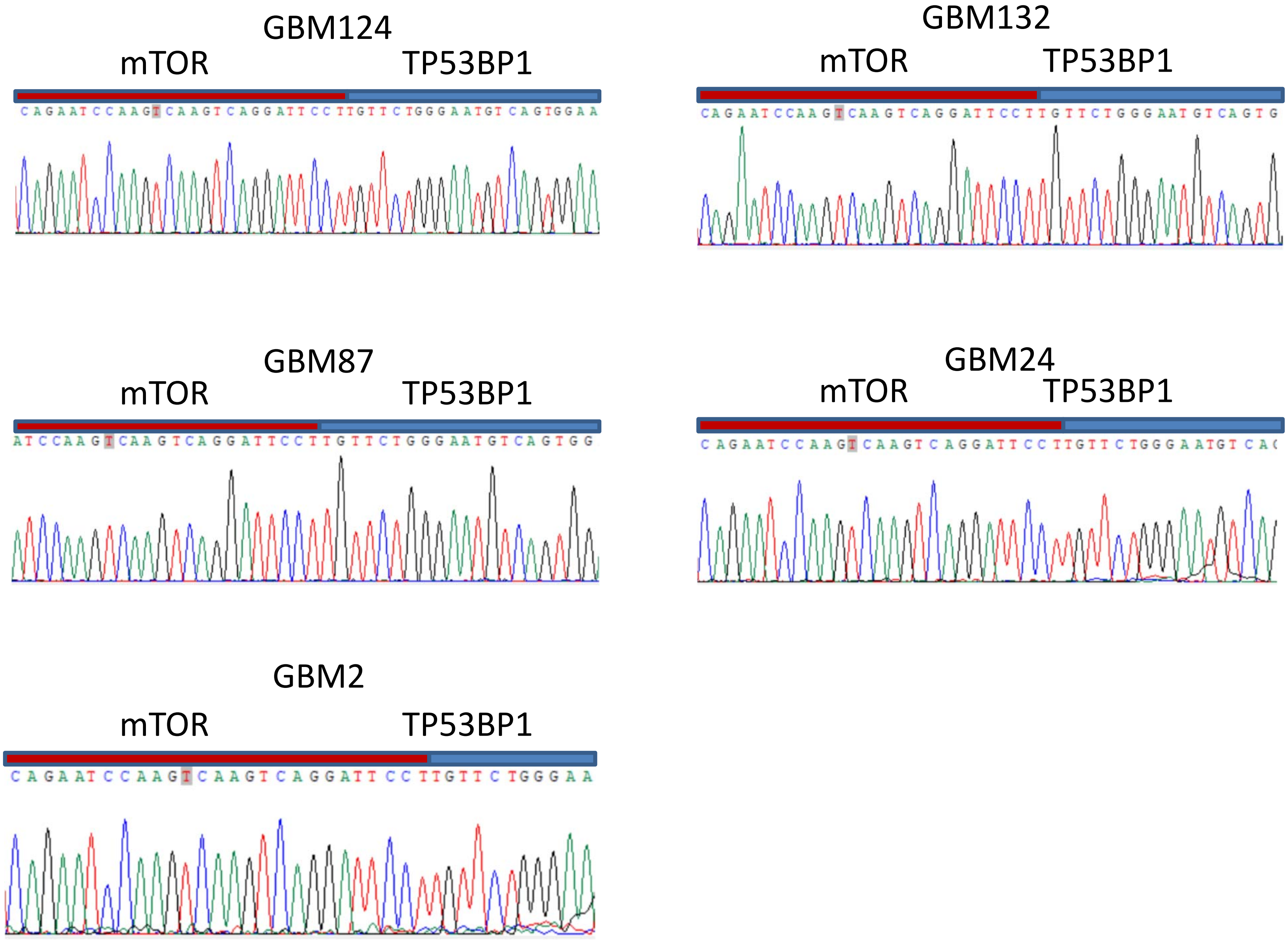
GBM124
mTOR
TP53BP1
CAGAATCCAAGTCAAGTCAGGATTCCTTGTTCTGGGAATGTCAGTGGAA
GBM132
mTOR
TP53BP1
CAGAATCCAAGTCAAGTCAGGATTCCTTGTTCTGGGAATGTCAGTG
GBM87
mTOR
TP53BP1
ATCCAAGTCAAGTCAGGATTCCTTGTTCTGGGAATGTCAGTGG
GBM24
mTOR
TP53BP1
CAGAATCCAAGTCAAGTCAGGATTCCTTGTTCTGGGAATGTCA
GBM2
mTOR
TP53BP1
CAGAATCCAAGTCAAGTCAGGATTCCTTGTTCTGGGAA

Supplemental figure 6.

KDM4-AC011523.2 fusion
Clinical specimens

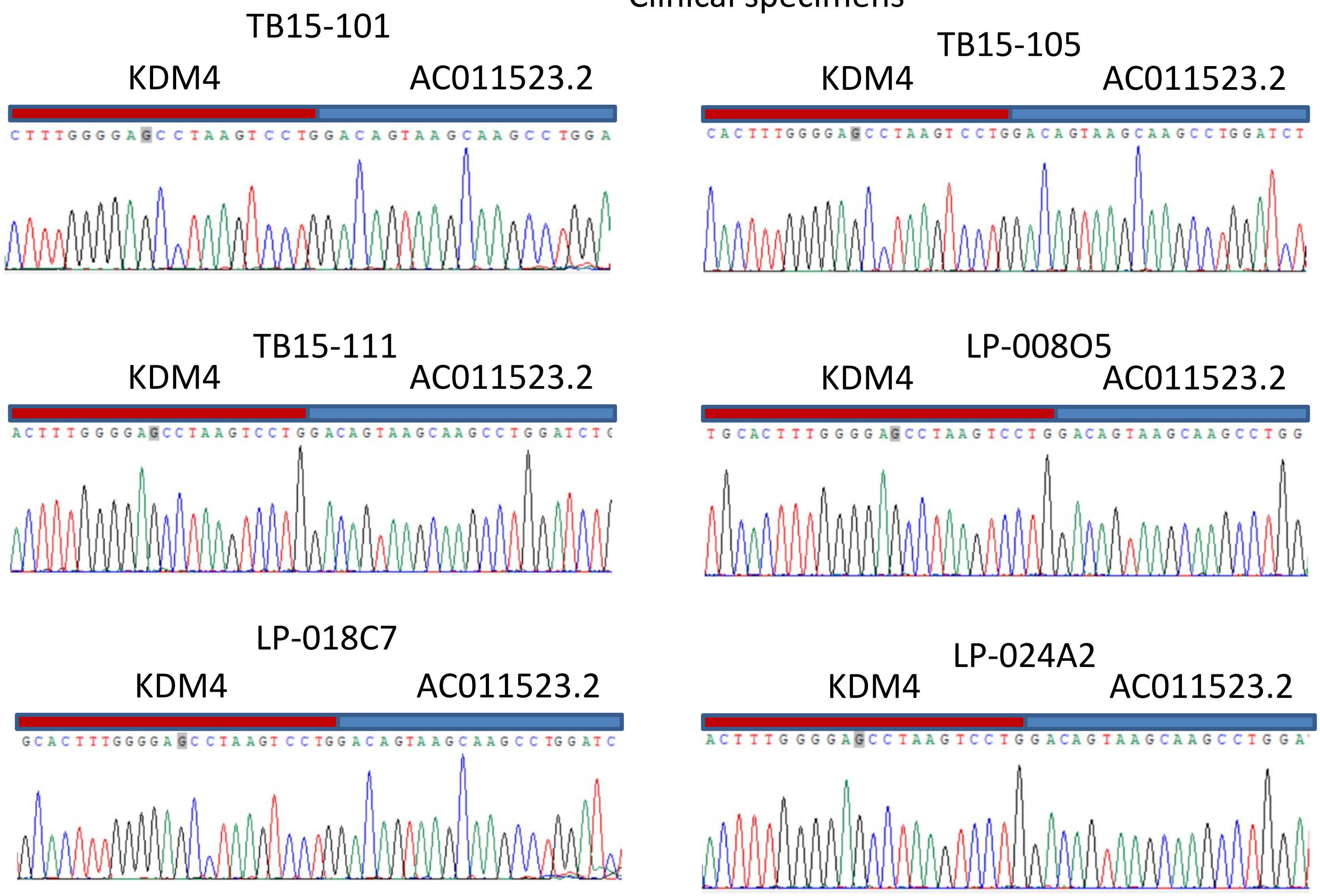

Supplement: Supplementary file 1 — Supplemental tables 1-7, supplemental figure legend and supplemental figures 1-6 [file 41598_2019_38550_MOESM1_ESM.pdf]
